# The paradox of pandemic mitigation? Moderating role of pandemic severity on the impact of social distancing policies: a cultural value perspective

**DOI:** 10.1186/s12992-024-01018-y

**Published:** 2024-02-09

**Authors:** Xingyang Ma, Bing Chen, Yufang Zhao

**Affiliations:** Faculty of Psychology, Southwest University, No.2 Tiansheng Road, Chongqing, Beibei China

**Keywords:** Social distancing policy, Mobility, Pandemic severity, Cultural values

## Abstract

**Background:**

Social distancing policies were of utmost importance during the early stages of the COVID-19 pandemic. These policies aimed to mitigate the severity of local outbreaks by altering public behavior. However, if the severity of the pandemic reduces, the impact of these policies on actual behavior may decrease. This study aims to examine, from a global perspective, whether the impact of social distancing policies on actual mobility is moderated by local pandemic severity and whether this moderating effect varies across cultural value contexts.

**Methods:**

We combined multiple publicly available global datasets for structural equation model analysis. 17,513 rows of data from 57 countries included in all databases were analyzed. Multilevel moderated moderation models were constructed to test the hypotheses.

**Results:**

More stringent policies in a region mean less regional mobility (*β* = -0.572, *p* < 0.001). However, the severity of local outbreaks negatively moderated this effect (*β* = -0.114, *p* < 0.001). When the pandemic was not severe, the influence of policy intensity on mobility weakened. Furthermore, based on Schwartz’s cultural values theory, cultural values of autonomy (*β* = -0.109, *p* = 0.011), and egalitarianism (*β* = -0.108, *p* = 0.019) reinforced the moderating effect of pandemic severity. On the other hand, cultural values of embeddedness (*β* = 0.119, *p* = 0.006) and hierarchy (*β* = 0.096, *p* = 0.029) attenuated the moderating effect.

**Conclusions:**

Social distancing policies aim to reduce the severity of local pandemics; however, the findings reveal that mitigating local pandemics may reduce their impact. Future policymakers should be alert to this phenomenon and introduce appropriate incentives to respond. The results also show that the moderating role of pandemic severity varies across cultures. When policies are promoted to deal with global crises, policymakers must seriously consider the resistance and potential incentives of cultural values.

**Supplementary Information:**

The online version contains supplementary material available at 10.1186/s12992-024-01018-y.

## Background

As the risk to human health from COVID-19 continues to decrease, on 5 May 2023, WHO declared the end of COVID-19 as a public health emergency of international concern (PHEIC) [1]. However, looking back at the early stages of the pandemic, we were faced with the virus without sufficient knowledge and medical interventions. At that time, social distancing policies significantly curbed the spread of the virus [2], preventing the overwhelming healthcare systems, and buying time for scientific research and vaccine development [3]. To prepare against future outbreaks, it’s crucial to learn from the current pandemic and improve our comprehension of public social distancing behavior for policy optimization.

Social distancing policies aim to curtail mobility, ultimately reducing the pandemic severity. However, a paradoxical challenge may arise as the pandemic mitigates: with reduced benefits for policy compliance, the public may spontaneously resume activities, thereby weakening the impact of such policies on mobility. This study examined whether pandemic severity moderated the impact of policy on actual mobility from a global perspective. Furthermore, the global implementation of social distancing policies is deeply influenced by cultural values [4, 5]. Cultural values can alter the benefits of policy compliance. As the severity of the pandemic lessens, they can either motivate people to persist in adhering to policies or exacerbate spontaneous rebound of mobility. This study also explored whether the moderating effect of pandemic severity varies across cultural contexts.

### Social distancing policy and mobility

Social distancing policies, including school closures, limits placed on large public spaces, and bans on in-restaurant dining were implemented globally during the COVID-19 pandemic [2]. A critical aspect of these policies is that they primarily rely on public compliance [6]. Ideally, as policies became more stringent, more places were restricted, thus, less regional mobility should be expected. Conversely, lower policy stringency should result in higher mobility. Recent research has demonstrated an association between policy stringency and mobility [7, 8]. However, the public frequently deviates from policy-guided behaviors [6, 9]. Even when governments enforce these measures, non-compliance still persists [10, 11], which could potentially complicate the association between policy stringency and mobility. Greater stringency policies do not necessarily reduce mobility.

From the perspective of the rational choice theory, public behavior depends on the trade-off between the benefits and costs of available options [12]. When the benefits of adhering to social distancing policies decrease, it is rational for individuals to reduce costly prevention behaviors correspondingly. Factors that reduce compliance benefits may weaken the effect of policies on mobility.

### The moderating role of pandemic severity

The mitigation of the pandemic severity may reduce the benefits of compliance. Previous studies have found that as pandemic severity decreases, public perception of infection risk declines [13, 14], resulting in reduced support for social distancing policies [15] and a decrease in policy-induced behaviors [16]. Viewing these phenomena through the lens of rational choice theory, pandemic severity mitigation devalues the benefits of policy compliance [17], consequently leading to shifts in attitudes and behaviors towards these policies. As the depreciation of policy-induced behaviors accumulates at the population level, the impact of social distancing policies on mobility is likely to decrease. In this study, we assumed that, although more stringent policies predicted less mobility, this association might be moderated by local pandemic severity.

Rational choice theory also suggests that culture shapes preferences and inclinations, thereby influencing how individuals trade-off the benefits and costs of decisions [18–20]. The impact of declining compliance benefits owing to mitigation of pandemic severity may be buffered in some cultural contexts, while becoming more pronounced in others.

### Moderating effect of pandemic severity under different cultural values

As a core aspect of culture, cultural values represent the prevailing societal ideals of what are considered good and desirable [21]. Cultural value directly influences support toward social distancing policies [22] and the governments implementing these policies [23]. Preference for social distancing policies offers additional compliance benefits. As the pandemic severity decreases, this preference buffered the impact of declining benefits. Conversely, resistance and distrust towards policies may exacerbate non-compliance. Cultural values also shape the risk perception [24]. When the severity of the pandemic decreases, overestimating risks weakens the extent of declining benefits, thus aiding in maintaining adherence. Conversely, underestimating risk may amplify the impact of decreasing pandemic severity.

**Fig. 1:**
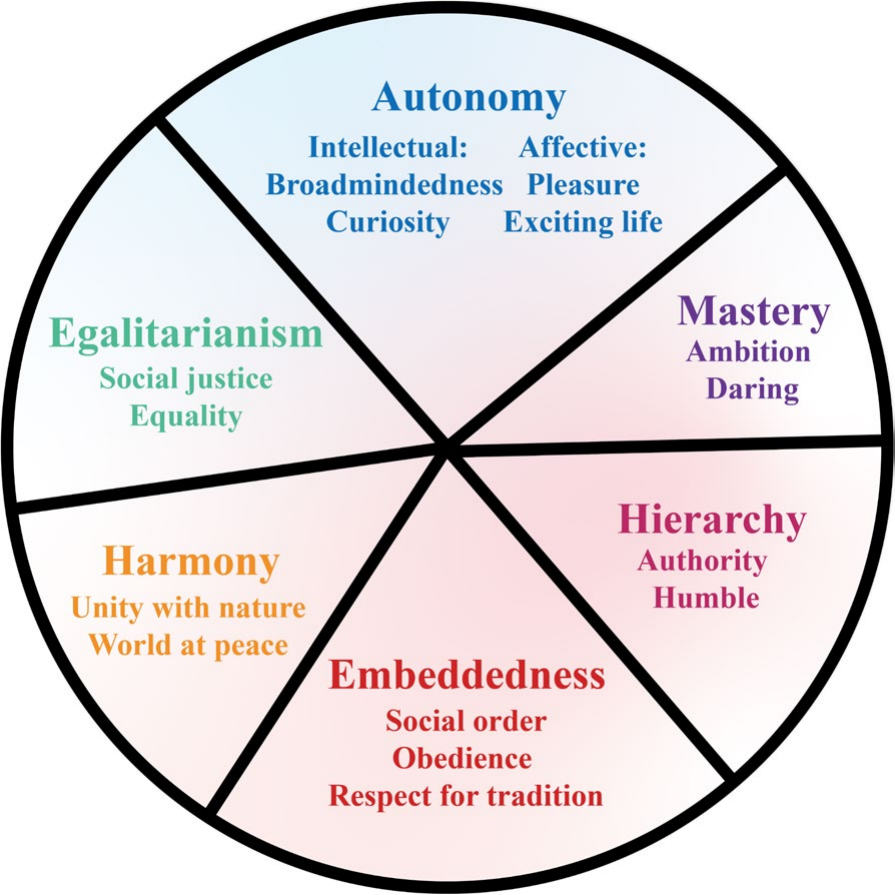
Structure of Schwartz’ cultural value theory

In the cross-cultural literature, Schwartz’s cultural value theory represents an innovative advance and has been examined in numerous empirical studies [25]. The primary characteristic of this theory is the simultaneous definition of distinct values and motivational conflicts between them [26]. As shown in Fig. 1, this theory proposes that societies’ opposite solutions to the three basic survival problems define three pairs of conflicting cultural values.

The first concerns the balance between individual autonomy and group integration, which defines Embeddedness as opposed to Autonomy. Embeddedness which relates to collectivism and uncertainty avoidance, emphasizes maintaining the status quo and avoiding actions that could disrupt group unity or order [27]. Embeddedness can both lead public to perceive greater risks [28, 29] and increase their support for social distancing policies [30]. As the severity of the pandemic decreases, these factors may buffer the impact of the declining benefits. Embeddedness may weaken the moderating effect of pandemic severity. Conversely, autonomy treats individuals as autonomous, bounded entities [26]. There are two forms of autonomy: intellectual autonomy, which overlaps with individualism, and affective autonomy, which aligns with uncertainty acceptance [27]. Autonomy implies reduced policy support [31] and a tendency to underestimate risk [24, 32]. As the pandemic severity decreases, these characteristics may exacerbate the depreciation of compliance benefits. In cultures that value autonomy, the public is more likely to spontaneously regain mobility as the severity of the pandemic decreases.

The second problem is guaranteeing that people behave in a responsible manner that preserves the social fabric, which defines Hierarchy versus Egalitarianism. Hierarchy emphasizes the legitimacy of a hierarchical social order. Social distancing behavior may be motivated by respect for and obedience to authority [33]. In societies that prioritize hierarchy, respect for authority may lead the public to be more supportive of policies formulated by public health experts or government officials [34]. As the severity of the pandemic diminishes, a preference for authority may lead to an increased inclination to continue following policy guidance. Conversely, egalitarianism emphasizes that all individuals in society are equal [26]. Within cultures that emphasize egalitarianism, the public may exhibit a greater tendency to question their leaders [35] and distrust the decisions made by government authorities [36]. In such cultures, the public may rely more on their own judgment of pandemic severity to make decisions, rather than authoritative guidance. Therefore, egalitarianism may enhance the moderating effect of pandemic severity.

The third problem is regulating people’s treatment of human and natural resources and defining Mastery versus Harmony. Mastery emphasizes achieving group or personal goals by mastering, directing, and altering the natural and social environment. Harmony emphasizes adapting to nature, accepting, preserving, and appreciating things as they are [26]. The value conflict between mastery and harmony might be prominently manifested in the debate regarding the notion of “coexisting with the virus”. Mastery values may incline individuals toward supporting proactive crisis intervention [37]. Social distancing policies are more likely to be supported by mastery. Such a preference for social distancing policies may lead to a more stable public policy compliance, rather than changing based on the severity of the pandemic. Conversely, the value of harmony expresses a contrasting strategy of adaptation. Less support for intervention measures and act according to severity situations seems to be more in line with the behavioral pattern associated with this value. The moderating effect of pandemic severity may be amplified by harmony values.

### Present research

Based on the above, we expected that more stringent policies could predict lower mobility in a country, but pandemic severity could moderate this association. As the local pandemic severity is mitigated, the relationship between policy stringency and actual mobility attenuates. Furthermore, we explored whether diverse cultural values moderated the moderating role of pandemic severity. We anticipate that the values of embeddedness, hierarchy, and mastery will buffer the moderating effect of pandemic severity mitigation on the link between policies and mobility. In contrast, the moderating effect may be enhanced in societies that prioritize autonomy, egalitarianism and harmony.

## Methods

### Variables and data sources

During the pandemic, global mobility and policy stringency data were shared. This study tested the model by combining these databases with existing databases of cultural values and other macro-control variables.

### Social distancing policy stringency.

We used data from the Oxford COVID-19 Government Response Tracker (OxCGRT) project [2]. The project tracks government policies and interventions across a standardized series of indicators and creates a suite of composite indices to measure the extent of these responses. The Government Stringency Index (GSI) is a composite indices that provides a systematic cross-national and cross-temporal measurement. The GSI on a given day indicates the degree of mobility restrictions implemented in a region. It ranges from 0 to 100, with higher score, indicating stricter policy measures are adopted.

### Mobility

The anonymized mobility dataset was provided by Google [38] for six locations: (1) retail and recreation, (2) grocery and pharmacy, (3) parks and outdoor spaces, (4) transit stations, (5) workplaces, and (6) residential areas. For indicators 1–5, a lower value indicates fewer visitors to the area compared to the baseline period. Indicator 6 reflects changes in the duration of time spent at home compared to the baseline level, with higher scores indicating that people spent more time at home.

### Pandemic severity

This study used daily new cases (DNC) as a measure of local pandemic severity [39]. The Center for Systems Science and Engineering at Johns Hopkins University provides the worldwide numbers of DNC per million people [40]. The number of daily new cases in different regions originated different data sources including official and unofficial sources.

### Schwartz’s cultural values

Schwartz and his collaborators collected data from 80 countries’ teacher and student samples and calculated cultural value orientation scores for each country [26]. According to Schwartz’s theory of cultural values, there are seven orientations, viz., (1) embeddedness, (2) intellectual autonomy, (3) affective autonomy, (4) hierarchy, (5) egalitarianism, (6) mastery, and (7) harmony. In this study, the two types of autonomy values were averaged to create a single autonomy score.

### Control variables

Regional macro characteristics are also related to mobility. Similar to previous studies, variables such as human development index (HDI) [41], population density (PD) [42], and 2021 Gross Domestic Product per capita (GDP) [43] were included as controls [44].

### Statistical analysis and procedure

This study uses data from repeated assessments conducted in different countries. Traditional regression models assume that all observations are independent and identically distributed, which can lead to biased estimates and incorrect conclusions regarding repeated datasets [45]. Multilevel regression models consider the dependencies between observations within the same country, allowing for a more accurate estimation of the effects of predictor variables on the outcome variables [46]. In this study, we used multilevel regression analysis to examine a moderated moderation model.

This study initially merges and filters data from various databases. Countries included in the GSI, Mobility, and Cultural Value databases were selected. In terms of time, this study focused on the early stages of the pandemic. For the endpoint of the study, we chose January 25, 2021, the day the first vaccine received emergency use validation from the WHO [47]. The development of a vaccine could impact the social distancing policy itself [48] and the public’s actual behavior [49]. This timing selection of data also avoids the potential impact of the vaccine policy. Additionally, considering the differences in mobility patterns between seasons, the chosen period also covered four seasons [50]. Ultimately, data from 57 countries, comprising 17,513 data rows, were included in our analysis. The mean and standard deviation of each variable are summarized by countries in the Supplementary Material (Online Supplementary Materials Table 1).

In our analysis, we initially employed factor analysis to consolidate multiple mobility categories into fewer composite indicators. We then examined the correlations between the variables at the national level. Next, we investigated the association between GSI and mobility. Subsequently, we assessed the moderating effect of the DNC on the relationship between the GSI and mobility. Finally, we incorporated various cultural values to test the moderated moderation models.

## Results

Exploratory factor analysis was used to reduce the number of mobility categories. The mean KMO of mobility at the six sites was 0.844, and the Bartlett’s sphericity test *p*-value was less than 0.001. Only one factor had an eigenvalue greater than 1, explaining 72.1 percent of the variance. The factor loadings of the different categories were as follows: Retail and recreation = 0.972, Grocery and pharmacy = 0.869, Transit stations = 0.906, Parks and outdoor spaces = 0.511, Workplaces = 0.826 and Residential areas = -0.928. The five categories with positive loads represented the number of visits to the corresponding public areas. We combined the averages of the standardized values of these categories into one indicator: public space (PS) mobility. A lower PS indicates lower mobility. Residential areas (RE), a negative factor load, also represents a distinct unit of measurement compared to other categories: average duration spent in places of residence. A lower RE indicates higher mobility. We separately constructed models using PS and RE as mobility indicators. Given that RE has an inverse relationship with mobility (higher RE means lower mobility), whereas PS is more intuitive (higher PS means higher mobility), we included the results of the PS models in the main text, while the detailed results and figures of the RE models are available in the online supplementary material.

### Correlational analyses at the national level

Table 1 summarizes the national-level correlations among variables. At the national level, there was no significant correlation between the GSI and mobility. Cultural values were also associated with mobility. Countries that valued autonomy had higher PS (r = 0.305, *p* = 0.021). Hierarchy values correlated with PS negatively (r = -0.415, *p* = 0.001). Harmony values correlated with PS positively (r = 0.271, *p* = 0.041).

**Table 1:** Pearson correlations of GSI, DNC, mobility, cultural values, and control variable

Variable	1	2	3	4	5	6	7	8	9	10	11	12	13
1 GSI	—												
2 DNC	0.073	—											
3 PS	0.121	0.036	—										
4 RE	-0.122	0.002	-0.810***	—									
5 Embeddedness	-0.111	-0.048	-0.256	0.163	—								
6 Autonomy	0.125	0.075	0.305*	-0.258	-0.935***	—							
7 Hierarchy	-0.245	-0.037	-0.415**	0.406**	0.606***	-0.557***	—						
8 Egalitarianism	0.111	-0.108	0.058	0.078	-0.579***	0.425***	-0.488***	—					
9 Mastery	-0.158	-0.037	-0.205	0.237	-0.050	0.067	0.288*	-0.164	—				
10 Harmony	0.020	-0.014	0.271*	-0.338*	-0.615***	0.508***	-0.592***	0.401**	-0.291*	—			
11 HDI	0.313*	-0.010	0.236	-0.208	-0.808***	0.824***	-0.597***	0.368**	-0.140	0.548***	—		
12 GDP	0.348**	-0.074	0.200	-0.192	-0.744***	0.755***	-0.581***	0.367**	-0.155	0.466***	0.958***	—	
13 PD	0.094	-0.147	-0.090	0.282*	0.041	-0.071	0.174	-0.043	-0.010	-0.134	0.145	0.217	—

*GSI* Government Stringency Index, *DNC* daily new cases, *PS* public space, *RE* residential area, *HDI* human development index, *GDP* gross domestic product, *PD* population density, DNC and GDP are based on logarithmic conversion data

^†^* p* < 0.01. * p < 0.05, ** p < 0.01, *** p < 0.001

### GSI-mobility multilevel analysis

As the first step in the multilevel model building process, the intraclass correlation (ICC) was computed in a null model. Data from different countries was divided into J clusters, indexed by j (j = 1…, J). Each cluster holds I units, indexed by i (i = 1…, I). The notations for the null model are as follows (model 1):

1 $$\begin{array}{l}\mathrm{Level}\;1:{\text{Mobility}}_{ij}=\beta_{0j}+r_{ij}\\\mathrm{Level}\;2:\beta_{0j}=\gamma_{00}+\mu_{0j}\end{array}$$

We omitted mobility and allowed intercepts to vary across countries to build a null model. The ICC of the PS model was greater than 0 (PS: ICC = 0.319). The subsequent multilevel model analysis was reasonable [51]. Subsequently, GSI and control variables were added. Referring to previous studies, GDP was converted into logarithms [50]. GSI was centralized within each country. The notations are as follows (model 2):

2 $$\begin{array}{ll} \text{Level}\;1:&{\text{Mobility}}_{ij}=\beta_{0j}+\beta_{1j}{GSI}_{ij}+r_j\\\text{Level}\;2:&\beta_{0j}=\gamma_{00}+\gamma_{01}{\text{GDP}}_j+\gamma_{02}{\text{HDI}}_j+\gamma_{03}{\text{PD}}_j+\mu_{0j}\\&\beta_{1j}=\gamma_{10}+\mu_{1j}\end{array}$$

This finding is consistent with the hypothesis that the GSI negatively predicts mobility (Online supplementary Table 2 for detailed results). A more stringent policy predicted PS negatively (*β* = -0.572, *p* < 0.001).

### Moderation effect of pandemic severity

Next, we examined whether the DNC moderated the relationship between GSI and mobility. The slopes of GSI, DNC and their interaction term on mobility were allowed to have random effects. We performed a log-transform of DNC [52]. The GSI and DNC were centralized within each country. The notations are as follows (model 3):

3 $$\begin{array}{ll}\mathrm{Level}\;1:&{\text{Mobility}}_{ij}=\beta_{0j}+\beta_{1j}{GSI}_{ij}+\beta_{2j}{DNC}_{ij}+\beta_{3j}\left({\text{GSI}}_{ij}\times{\text{DNC}}_{ij}\right)+r_{ij}\\\mathrm{Level}\;2:&\beta_{0j}=\gamma_{00}+\gamma_{01}{\text{GDP}}_j+\gamma_{02}{\text{HDI}}_j+\gamma_{03}{PD}_j+\mu_{0j}\\&\beta_{1j}=\gamma_{10}+\mu_{1j}\\&\beta_{2j}=\gamma_{20}+\mu_{2j}\\&\beta_{3j}=\gamma_{30}+\mu_{3j}\end{array}$$

**Fig. 2:**
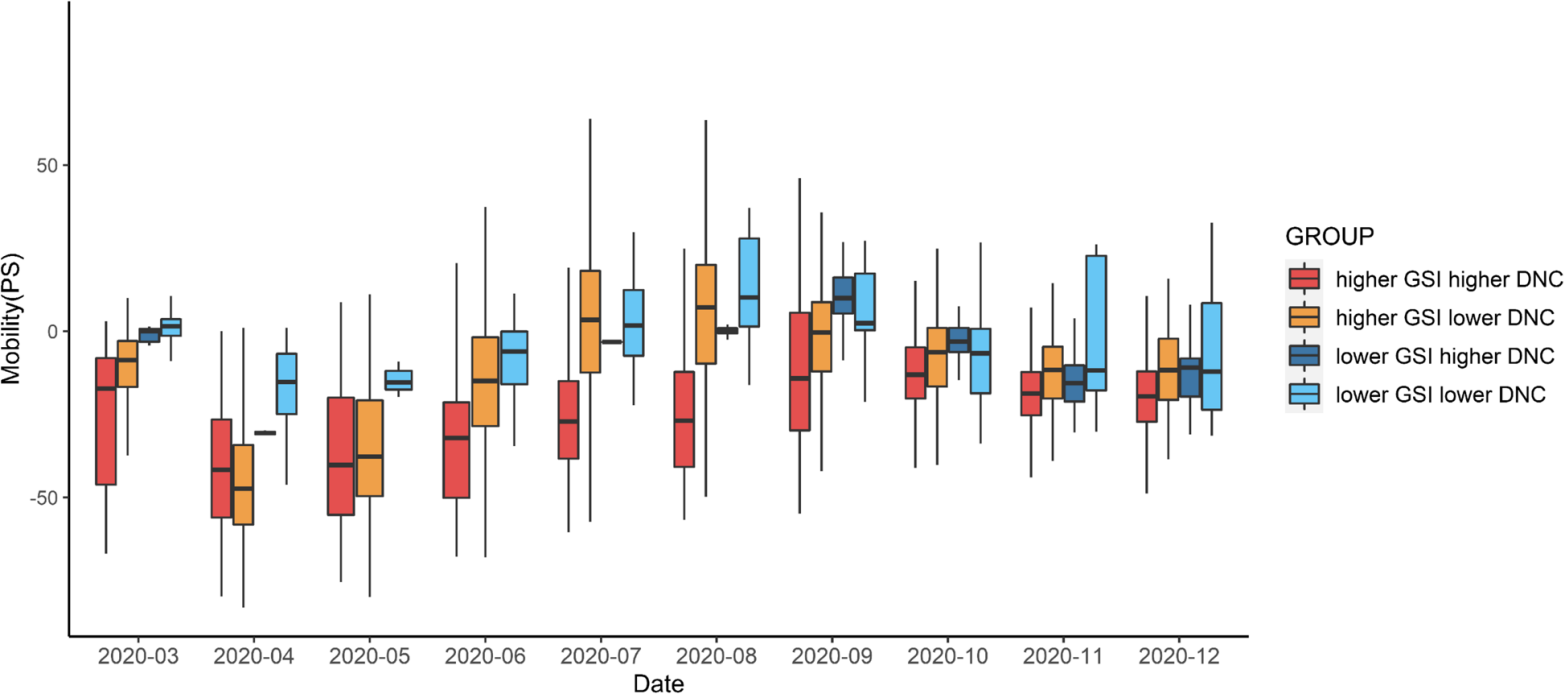
Conditional effect of GSI on PS as a function of DNC

As expected, the association between GSI and PS was stronger when there were more DNC (*β* = -0.114, *p* < 0.001) (Online supplementary Table 3 for the details). We also employed the Johnson-Neyman (J-N) technique [53] to identify the region of significance and the slope change trend of the conditional effect of GSI on mobility. In Fig. 2, as the DNC increased, the negative relationship between the GSI and PS became stronger.

**Fig. 3:**
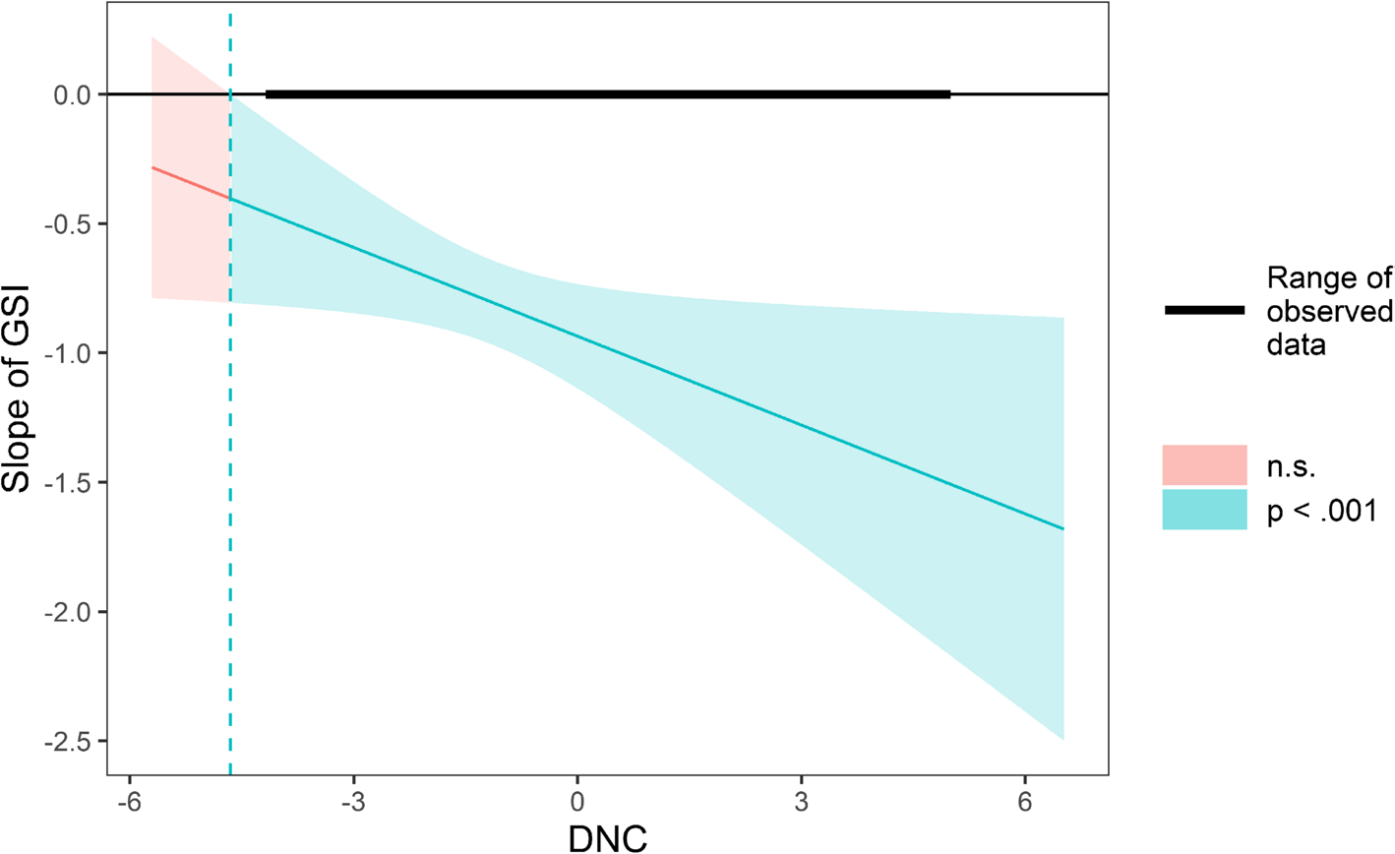
Average PS under GSI and DNC conditions in different periods

To better explain the interaction between the GSI and DNC on PS, we calculated the actual mobility in different GSI and DNC situations (higher GSI, lower GSI × higher DNC, and lower DNC) over different periods (refer to Fig. 3). At the same GSI, a higher DNC resulted in a lower PS. In the scenario of high GSI and high DNC, PS was the lowest; with high GSI and low DNC, PS was the second lowest; for low GSI and high DNC, PS is the third lowest; and for low GSI and low DNC, PS was the highest.

### The moderation of cultural values

We added the third-order interaction term (GSI × DNC × Value) to constructed the multilevel moderated moderation model. Cultural values were centralized among countries, and the GSI and DNC were also centralized within countries. The control variables were also handled as before. The notations are as follows (model 4):

4 $$\begin{array}{ll}{\text{Level}}\;1:& {{\text{Mobility}}}_{ij}={\beta }_{0j}+{\beta }_{1j}{{\text{GSI}}}_{ij}+{\beta }_{2j}{{\text{DNC}}}_{ij}+{\beta }_{3j}\left({{\text{GSI}}}_{ij}\times {{\text{DNC}}}_{ij}\right)+{r}_{ij}\\ {\text{Level}}\;2:& {\beta }_{0j}={\gamma }_{00}+{\gamma }_{01}{{\text{GDP}}}_{j}+{\gamma }_{02}{{\text{HDI}}}_{j}+{\gamma }_{03}{PD}_{j}+{\gamma }_{04}{{\text{Value}}}_{j}+{\mu }_{0j}\\ & {\beta }_{1j}={\gamma }_{10}+{\gamma }_{11}{{\text{Value}}}_{j}+{\mu }_{1j}\\ & {\beta }_{2j}={\gamma }_{20}+{\gamma }_{21}{{\text{Value}}}_{j}+{\mu }_{2j}\\ & {\beta }_{3j}={\gamma }_{30}+{\gamma }_{31}{{\text{Value}}}_{j}+{\mu }_{3j}\end{array}$$

Table 2 reports the standardized coefficients with the *p*-value of the GSI and interaction terms on PS. The three-way interaction terms of models of embeddedness (*β* = 0.119, *p* = 0.006) and hierarchy (*β* = 0.096, *p* = 0.029) were positively significant. In the models of autonomy (*β* = -0.109, *p* = 0.011), and egalitarianism (*β* = -0.108, *p* = 0.019), the terms were negatively significant. Embeddedness and hierarchy values weaken DNC's moderating effect, while autonomy and egalitarianism enhance it. However, mastery and harmony have no impact on DNC's moderating effect.

**Table 2:** Moderated moderation model of PS (the coefficients of the interaction terms)

Variables	Cultural Values					
	**Embeddedness**	**Autonomy**	**Hierarchy**	**Egalitarianism**	**Mastery**	**Harmony**
**GSI**	-0.638***	-0.683***	-0.682***	-0.682***	-0.681***	-0.682***
GSI*DNC	-0.171***	-0.170***	-0.170***	-0.170***	-0.168***	-0.170***
GSI*VALUE	0.094*	-0.091*	0.065	-0.074^†^	-0.097*	-0.062
DNC*VALUE	0.110**	-0.105**	0.116**	-0.081*	0.096*	-0.096*
GSI*DNC*VALUE	0.119**	-0.109*	0.096*	-0.108*	0.005	-0.073

*GSI* Government Stringency Index, *DNC* daily new cases

^†^ *p* < 0.01. * *p* < 0.05, ** *p* < 0.01, *** *p* < 0.001

**Fig. 4:**
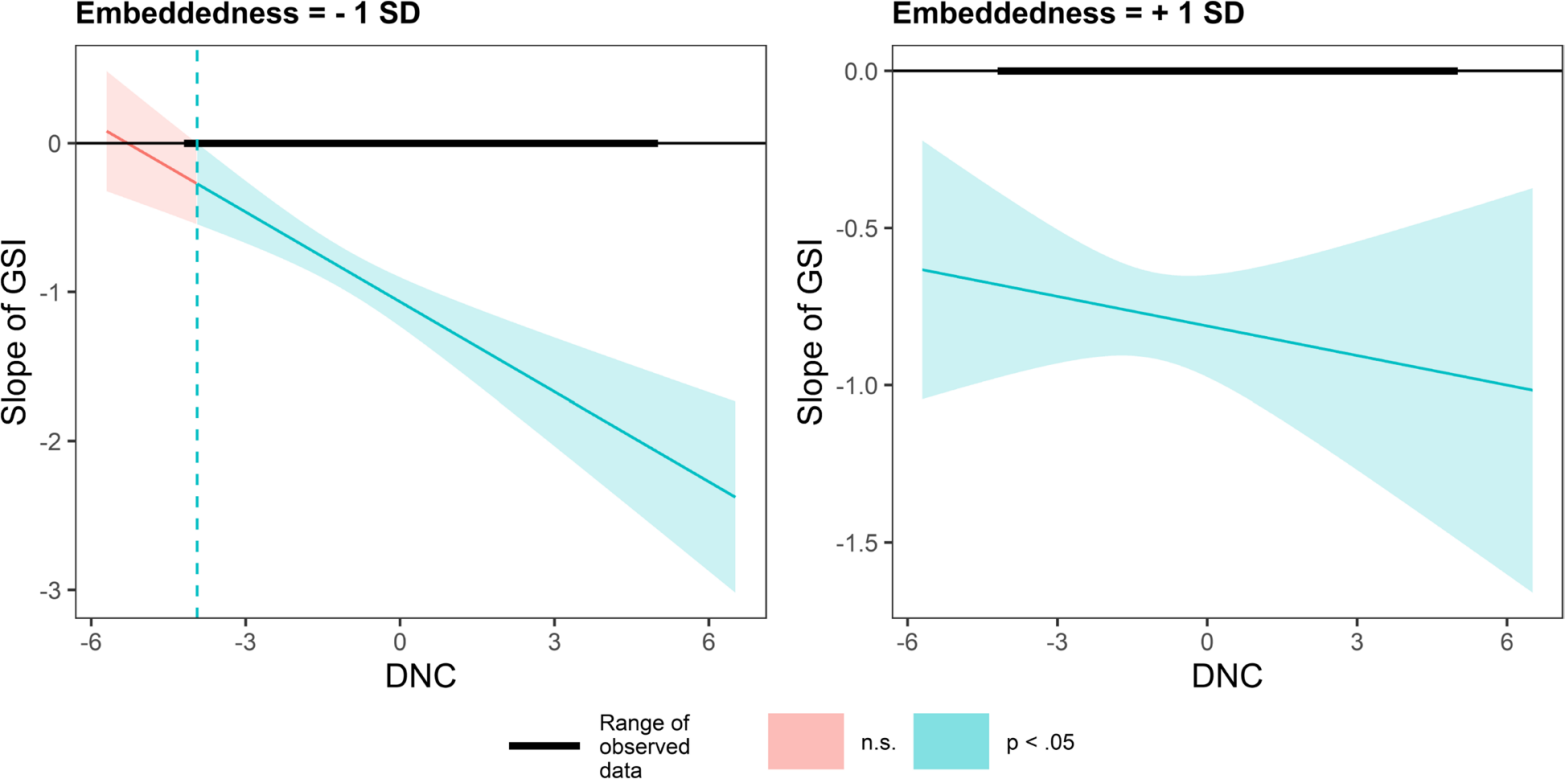
Conditional effect of GSI on PS as a function of DNC under different embeddedness values

**Fig. 5:**
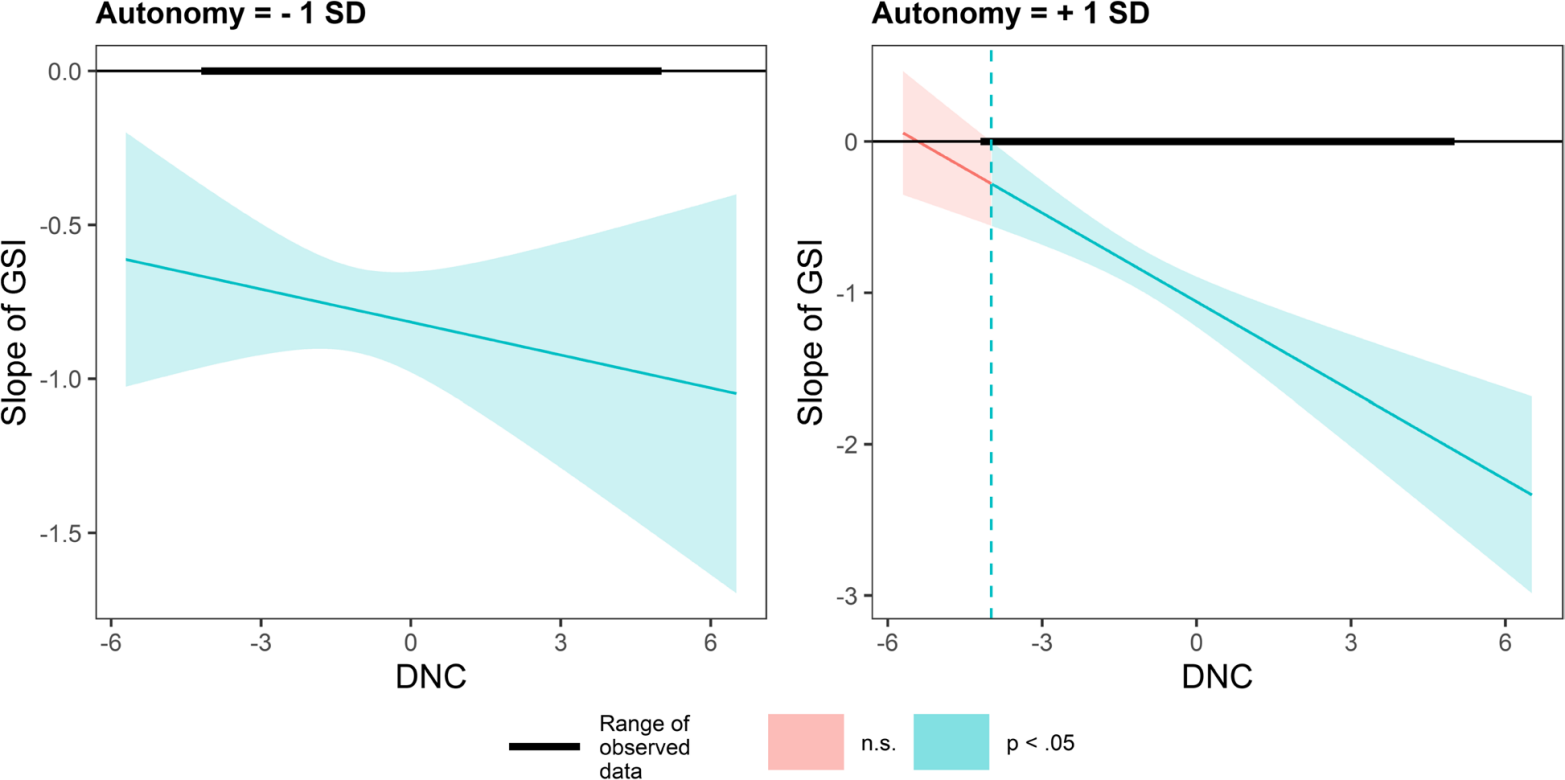
Conditional effect of GSI on PS as a function of DNC under different autonomy values

Using the Johnson-Neyman (J-N) technique, we plot the conditional effect of GSI on PS as a function of DNC under different values (mean ± 1SD). In Fig. 4, in countries valuing embeddedness, DNC has a smaller impact on GSI slope. In contrast, in countries valuing autonomy, DNC has a greater impact on the GSI slope (Fig. 5).

**Fig. 6:**
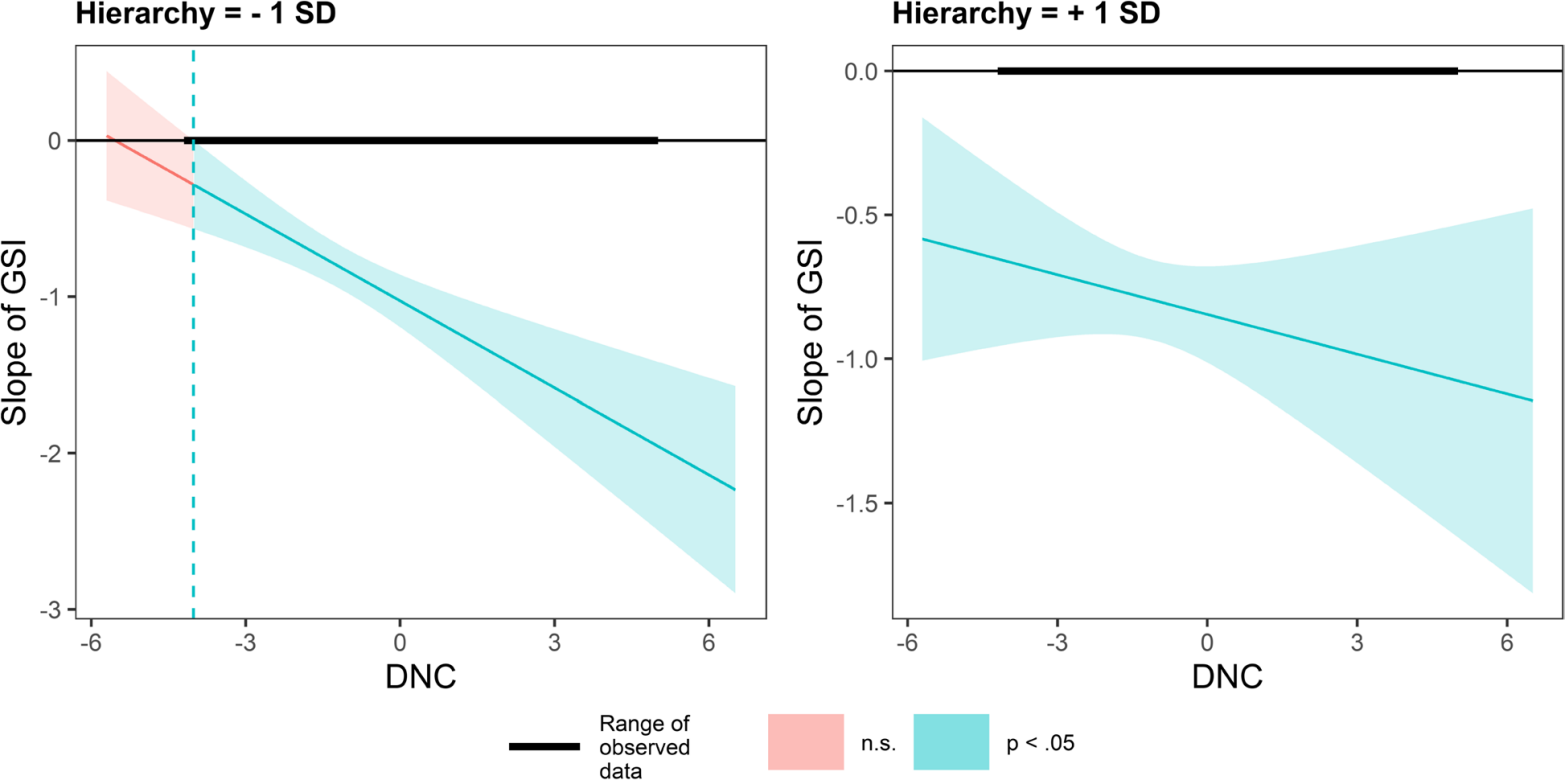
Conditional effect of GSI on PS as a function of DNC under different hierarchy values

**Fig. 7:**
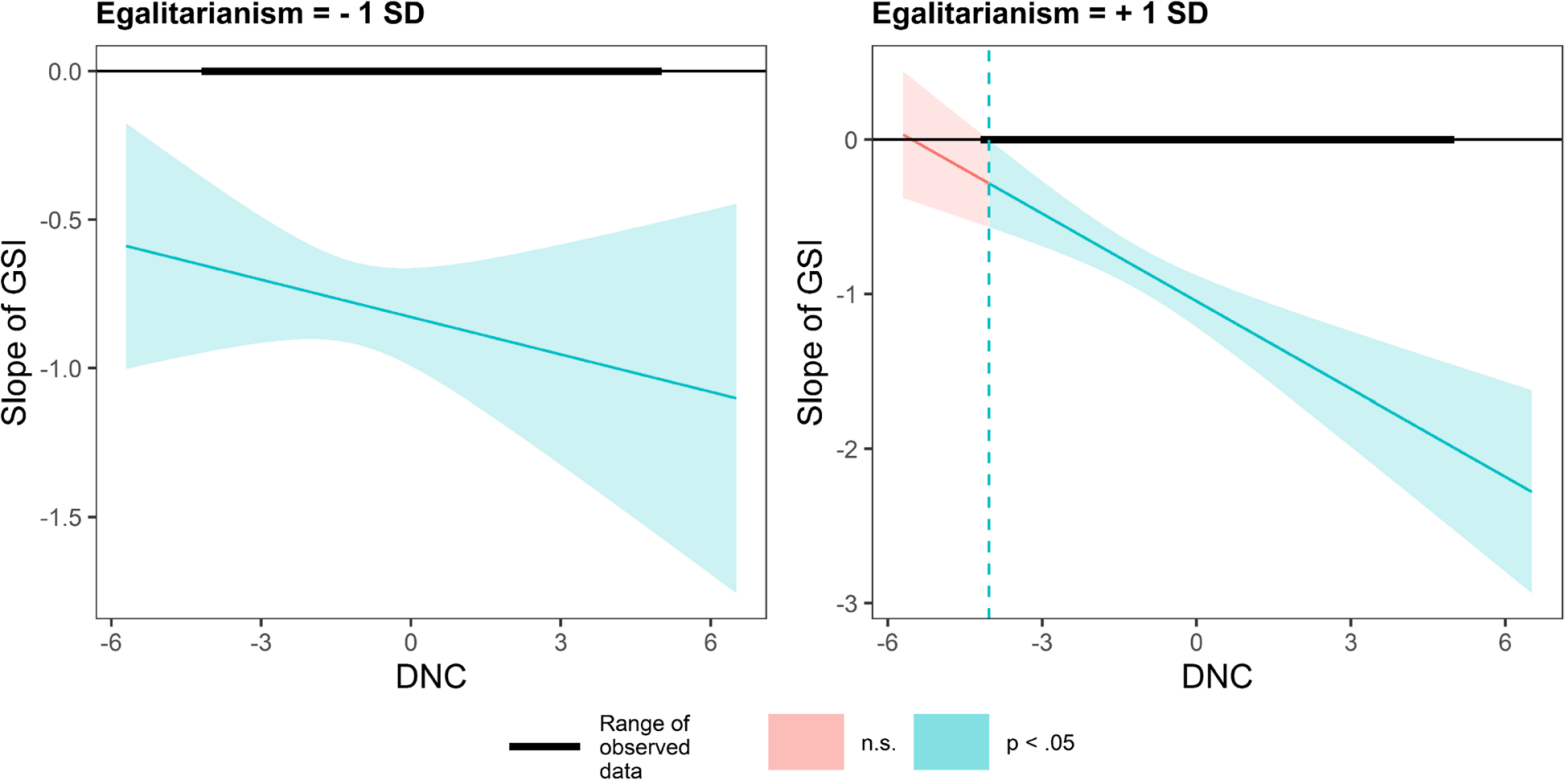
Conditional effect of GSI on PS as a function of DNC under different egalitarianism values

In Fig. 6, in countries valuing hierarchy, DNC has a smaller impact on GSI slope. In contrast, in countries valuing egalitarianism, DNC has a greater impact on the GSI slope (Fig. 7).

**Fig. 8:**
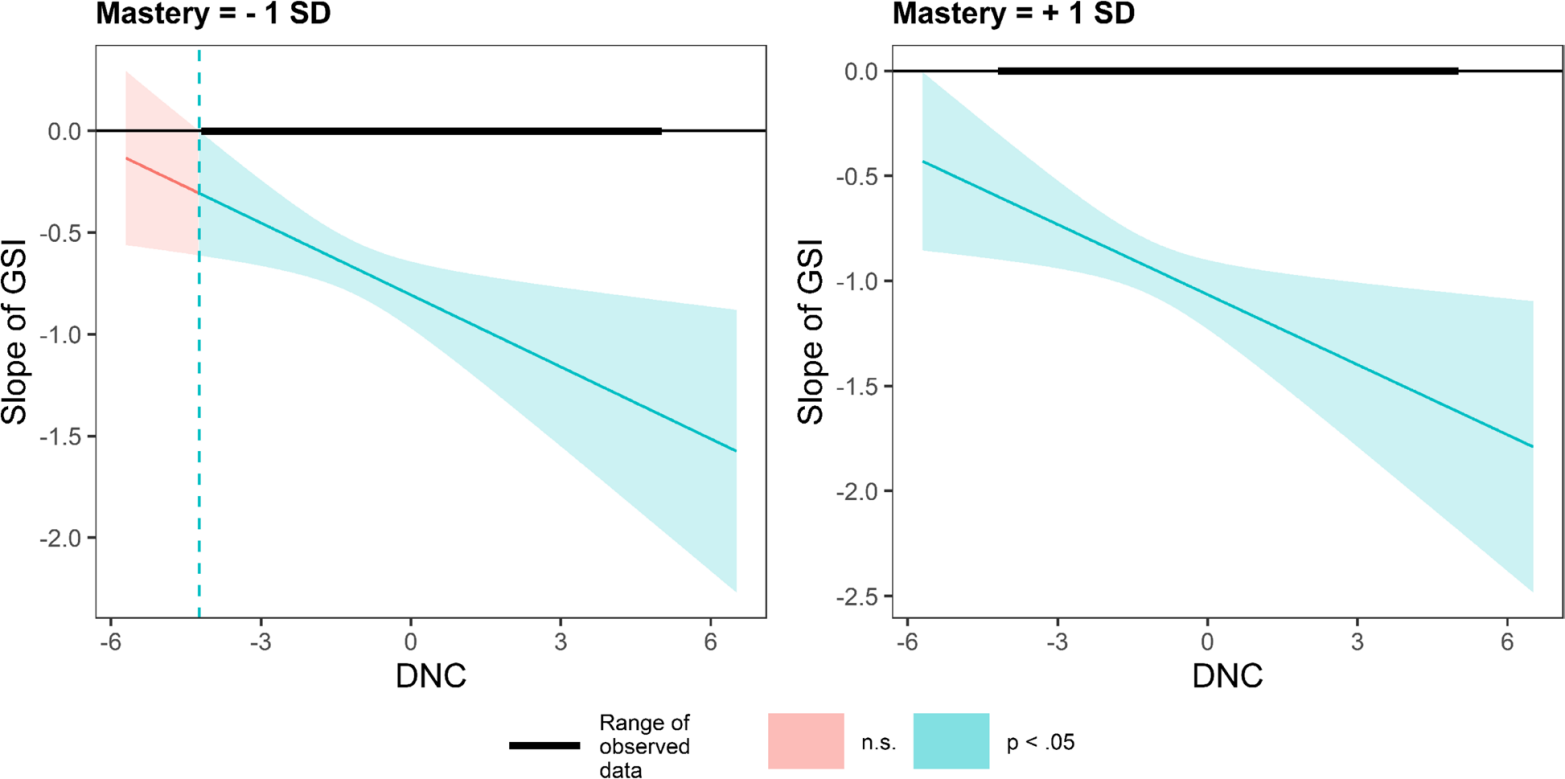
Conditional effect of GSI on PS as a function of DNC under different harmony values

**Fig. 9:**
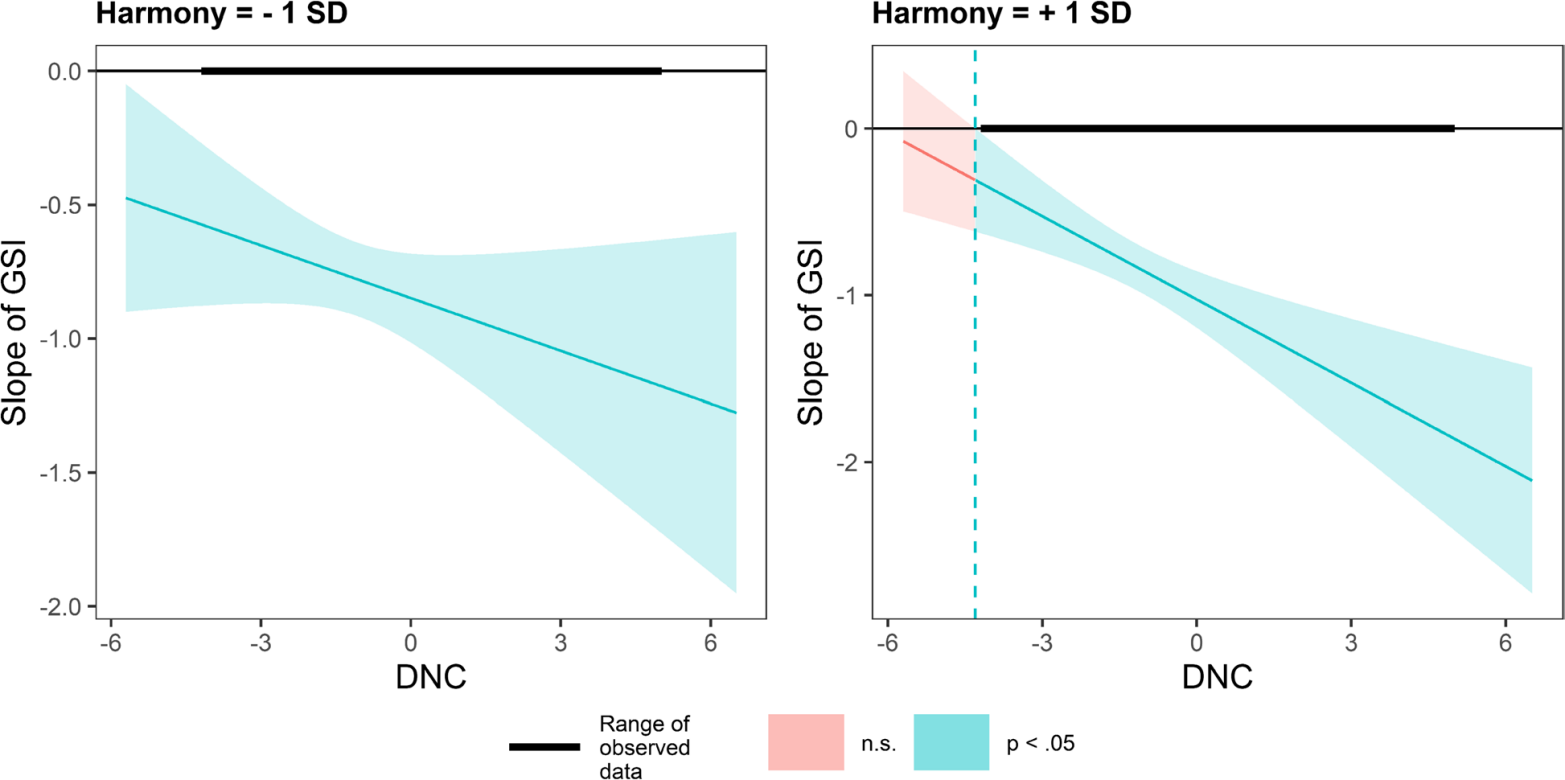
Conditional effect of GSI on PS as a function of DNC under different harmony values

In countries with varying degrees of emphasis on mastery (Fig. 8) and harmony (Fig. 9), there is no difference in the impact of DNC on the GSI slope.

To better comprehend the role of cultural values, as a supplementary analysis, we categorized countries into three equal segments based on their values and assessed the multilevel moderating effect of the DNC in countries with higher and lower values. In countries that prioritize embeddedness, the moderating effect is not significant (*β*_GSI*DNC_ = 0.002, *p* = 0.115). In countries that do not prioritize embeddedness, the moderating effect is significant (*β*_GSI*DNC_ = -0.378, *p* < 0.001). The effect of hierarchy and mastery is similar to that of embeddedness. Higher hierarchy: *β*_GSI*DNC_ = -0.157, *p* = 0.130; Lower hierarchy: *β*_GSI*DNC_ = -0.354, *p* < 0.001. Higher mastery: *β*_GSI*DNC_ = -0.166, *p* = 0.911; Lower mastery: *β*_GSI*DNC_ = -0.148, *p* = 0.017. In countries that prioritize autonomy, the moderating effect is significant (*β*_GSI*DNC_ = -0.356, *p* < 0.001). In countries that do not prioritize autonomy, the moderating effect is insignificant (*β*_GSI*DNC_ = -0.006, *p* = 0.965). The effect of egalitarianism and harmony is similar to that of autonomy values. Higher egalitarianism: *β*_GSI*DNC_ = -0.366, *p* < 0.001; Lower egalitarianism: *β*_GSI*DNC_ = -0.131, *p* = 0.296. Higher harmony:* β*_GSI*DNC_ = -0.283, *p* < 0.001; Lower harmony: *β*_GSI*DNC_ = -0.088, *p* = 0.400.

## Discussion

In the initial stages of the pandemic, social distancing policies effectively slowed the spread of the virus, providing valuable time for viral research and vaccine development. However, not all individuals consistently follow these policies. This study found that, from a global perspective, more stringent policies predicted reduced mobility, but this relationship was moderated by local pandemic severity. When pandemic severity declined, the association between policy stringency and mobility weakened. This suggests that, although the policies remained stringent, people may have spontaneously regained their mobility, as the severity of the pandemic was mitigated. This behavior aligns with the rational choice theory perspective, as a mitigated local pandemic means a lower infection risk and a decrease in the subjective benefits of policy-induced behavior. Nevertheless, prematurely resuming mobility may raise the probability of recurrent outbreaks [54], thereby jeopardizing long-term and collective interests. Policymakers and the public should remain vigilant and responsive to this “social distancing relaxation” accordingly. For instance, increased publicity about the policy or more incentives for compliance are required as risk is reduced. Our finding also highlights the importance of timely policy adjustments. Maintaining a policy that no one follows means nothing, but greater economic burden and greater divergence from public opinion.

Furthermore, our study revealed that the moderating effect of pandemic severity can be weakened by embeddedness and hierarchy, but enhanced by autonomy and egalitarianism. Embeddedness and hierarchy provide compensatory benefits, motivating the public to maintain policy compliance as the pandemic severity decreases. By contrast, autonomy and egalitarianism empowered individuals to rely on their own judgments regarding the situation, strengthening the moderating impact of pandemic severity. However, the pair of values, mastery and harmony, did not alter the moderating effect of pandemic severity. Mastery may promote support for social distancing policies, but is also conceptually linked to masculinity [27], which is associated with a higher risk tolerance [55]. Similarly, harmony may not favor proactive intervention measures, but this value is also associated with stronger moral obligation to reduce COVID-19 risk [56]. The contradictory nature of these values regarding social distancing may explain their inability to affect the moderation of pandemic severity. These findings confirmed the idea that cultural factors are crucial for the success of social distancing policies [4, 5]. Cultural values can be invisible but are ubiquitous “incentive” for policy adherence. To promote compliance, policies should be developed and implemented in a way that matches prevailing cultural values. Adjusting the values contained in the narrative of policy publicity has application prospect [57]. Customized policy information can promote compliance, for example, “adherence to the policies can protect the health of the family (embeddedness),” “can protect the lives of vulnerable groups (egalitarianism).”

## Theoretical contributions

This study expands our understanding of bounded human rationality in health-related choices. People are good at making decisions independently [58]. However, with bounded rationality, in the face of uncertainty and risk, we tend to make choices based on our feelings or intuition [59]. Noncompliance exposes an individual to the risk of infection, and in the long-term interests of the community, it also creates more serious problems such as repeated outbreaks [54] and health inequalities [60]. When the policy is not relaxed, they choose to relax their behavior, which may harm the interests of others, especially vulnerable groups.

Moreover, in the second strand of behavioral economics, the decision-maker is considered an enculturated actor [18]. Culture and rationality should be integrated into an explanation of public behavior. Traditional rational choice theory does not provide a good explanation for why people follow social norms of behavior that override personal interests to act selflessly and responsibly. Cultural values are considered part of the “common psychological currency” [61] that directly shapes how individuals calculate the costs and benefits of different actions [62]. Our findings indicate specific cultural values contribute to greater participation in social distancing policies, demonstrating that despite cognitive limitations, social factors, such as cultural values, can drive human behavior beyond individual interests. The theoretical perspective of culturally rational decision-makers enhances traditional theories and provides deeper insights into the complex and diverse range of human behaviors.

## Limitations and future directions

Future research can expand on different scales.

Firstly, regarding temporal scale, this study focused on the early stages of the pandemic, particularly before effective pharmaceutical interventions became available. However, the confounding factors were not fully addressed during the same period. Mask-wearing and social distancing were implemented simultaneously to complement each other, yet mask usage may magnify unrealistic risk optimism [63] and lead to risk compensation, resulting in reduced social distancing [64]. As the severity of the pandemic decreases, mask usage may potentially prompt spontaneous recovery in mobility. Future research should delve into the potential interference among policies. Over time and with the unfolding of new events, the benefits and costs of policies may change. For example, the emergence of benign virus variants or effective medical interventions could reduce mortality [65], thus reducing the benefits of maintaining social distancing. Additionally, with the continued implementation of these policies, the phenomenon of “social distancing fatigue” may become even more pronounced [66]. This can increase the policy compliance costs. As benefits decrease and costs increase, adherence to policies may become more challenging. Future research should examine the characteristics of policy compliance at different stages.

Secondly, regarding spatial scale, our study was conducted at the national level. Nonetheless, there are substantial cultural differences in protective behaviors between communities and regions within a country [67, 68], and the influence of political ideology transcends physical space [69]. Regions characterized by greater cultural diversity may pose greater challenges in policy management. Apart from the increased difficulty in achieving policy consensus in these regions, this study also implies that they may face a greater risk of outbreaks resurgence due to communities resuming mobility before policy relaxation is deemed appropriate. Further understanding of the role of cultural values in the ineffectiveness of policies in certain regions or communities is highly beneficial for domestic policymakers.

Thirdly, individual-level research provides a psychological foundation to explain macro-level phenomena. Numerous factors influence public perceptions of reality. For instance, during the COVID-19 pandemic, information overload is a potential influencing factor [70], which could lead individuals to either underestimate or overestimate risks [71]. Future research should explore the link between individual risk perception and the actual pandemic severity, as well as how this connection influences subsequent social distancing behaviors. The buffering effect of values on the mitigation of pandemic severity requires individual-level studies. Personal values may influence an individual's risk perception or inclination to comply with rules and norms. Future research should gather samples to probe into how values operate at the individual level.

## Conclusion

This study, conducted from a global perspective, revealed that lower pandemic severity leads to a reduced impact of social distancing policies on actual mobility. While social distancing policies are designed to reduce the severity of the pandemic, a mitigated pandemic severity may paradoxically weaken the impact of these policies. It is essential to remain vigilant about this phenomenon and introduce incentive measures at appropriate time to strengthen policy compliance. Furthermore, this study found that the cultural values of embeddedness and hierarchy weakened the moderating effect of pandemic severity on policy impacts on mobility. Conversely, autonomy and egalitarianism enhanced this moderating effect. Policymakers should be aware of both the motivating potential of cultural values in promoting policy compliance and the challenges posed by regional cultural attributes and differences.

### Supplementary Information

**Additional file 1: Supplementary Material. Table 1.** Descriptive statistics for study variables in the 57 countries. **Table 2.** Multilevel regression results for GSI on mobility. **Table 3.** Multilevel regression results for GSI*DNC on mobility. **Figure 1.** Conditional effect of GSI on mobility (RE) as a function of DNC. **Figure 2.** Average RE under GSI and DNC conditions in different periods. **Table 4.** Moderated moderation model of RE (the coefficients of the interaction terms). **Figure 3.** Conditional effect of GSI on RE as a function of DNC under different embeddedness values. **Figure 4.** Conditional effect of GSI on RE as a function of DNC under different autonomy values. **Figure 5.** Conditional effect of GSI on RE as a function of DNC under different hierarchy values. **Figure 6.** Conditional effect of GSI on RE as a function of DNC under different egalitarianism values. **Figure 7.** Conditional effect of GSI on RE as a function of DNC under different mastery values. **Figure 8.** Conditional effect of GSI on RE as a function of DNC under different harmony values.

**Additional file 2.**

**Additional file 3.**

**Additional file 4.**

**Additional file 5.**

## Data Availability

This study integrates several publicly available databases that can be downloaded according to the data source if needed. The data and code used this study are available upon request.

## Abbreviations

COVID-19: Coronavirus disease 2019
SD: Standard deviation
WHO: World Health Organization
GSI: Government stringency index
DNC: Daily new cases
OxCGRT: Oxford COVID-19 Government Response Tracker
HDI: Human development index
GDP: Gross Domestic Product per capita
PD: Population density
