# Supplementary material for: The paradox of pandemic mitigation? Moderating role of pandemic severity on the impact of social distancing policies: a cultural value perspective
Source: Global Health. 2024 Feb 9;20:13. doi: 10.1186/s12992-024-01018-y (PMC10854019; doi:10.1186/s12992-024-01018-y)
Supplement: Supplementary file 1 — Additional file 1: Supplementary Material. Table 1. Descriptive statistics for study variables in the 57 countries. Table 2. Multilevel regression results for GSI on mobility. Table 3. Multilevel regression results for GSI*DNC on mobility. Figure 1. Conditional effect of GSI on mobility (RE) as a function of DNC. Figure 2. Average RE under GSI and DNC conditions in different periods. Table 4. Moderated moderation model of RE (the coefficients of the interaction terms). Figure 3. Conditional effect of GSI on RE as a function of DNC under different embeddedness values. Figure 4. Conditional effect of GSI on RE as a function of DNC under different autonomy values. Figure 5. Conditional effect of GSI on RE as a function of DNC under different hierarchy values. Figure 6. Conditional effect of GSI on RE as a function of DNC under different egalitarianism values. Figure 7. Conditional effect of GSI on RE as a function of DNC under different mastery values. Figure 8. Conditional effect of GSI on RE as a function of DNC under different harmony values. [file 12992_2024_1018_MOESM1_ESM.docx]

**Supplementary Material：**

| Table 1 Descriptive statistics for study variables in the 57 countries | | | | | | | | | | | | | | | | | |
| --- | --- | --- | --- | --- | --- | --- | --- | --- | --- | --- | --- | --- | --- | --- | --- | --- | --- |
| location | GSI | DNC | RR | GP | RE | TS | PA | WO | HDI | GDP ($1000) | PD | MA | HA | EM | AU | HI | EG |
| Austria | 55.70  (19.77) | 128.98  (208.36) | -31.35  (26.05) | -12.35  (10.89) | 7.32  (7.17) | -31.89  (18.76) | 24.12  (48.22) | -29.17  (14.7) | 0.92 | 45.44 | 106.75 | 3.92 | 4.31 | 3.11 | 4.60 | 1.75 | 4.89 |
| Belgium | 58.14  (17.32) | 173.23  (316.25) | -31.78  (24.06) | -8.65  (9.24) | 11.55  (7.11) | -34.06  (18.38) | 29.03  (42.29) | -31.23  (15.95) | 0.93 | 42.66 | 375.56 | 3.84 | 4.35 | 3.25 | 4.29 | 1.69 | 5.20 |
| Bolivia | 82.67  (15.41) | 46.11  (45.01) | -51.77  (21.95) | -36.49  (24.87) | 21.36  (10.76) | -50.44  (23.35) | -45.38  (21.19) | -37.18  (22.83) | 0.72 | 6.89 | 10.20 | 3.87 | 4.11 | 4.07 | 3.53 | 2.66 | 4.74 |
| Brazil | 67.16  (15.72) | 116.17  (83.01) | -32.44  (17.16) | 4.56  (12.96) | 10.47  (4.8) | -27.88  (17.6) | -32.54  (15.21) | -13.74  (13.8) | 0.77 | 14.10 | 25.04 | 3.93 | 4.03 | 3.62 | 3.90 | 2.37 | 4.89 |
| Bulgaria | 48.35  (13.39) | 97.66  (165.7) | -20.21  (21.96) | -3.92  (10.42) | 3.99  (6.99) | -18.46  (19.85) | 27.01  (45.9) | -24.63  (11.42) | 0.82 | 18.56 | 65.18 | 4.02 | 4.13 | 3.87 | 3.88 | 2.68 | 4.13 |
| Cameroon | 50.75  (16.99) | 3.23  (7.59) | -7.74  (8.6) | -0.82  (10.54) | 5.23  (3.67) | 7.30  (18.36) | -14.18  (6.8) | -10.13  (5.38) | 0.56 | 3.36 | 50.89 | 3.60 | 4.19 | 4.46 | 2.86 | 2.46 | 4.68 |
| Canada | 62.98  (18.08) | 49.94  (57.57) | -23.00  (15.68) | -4.19  (9.08) | 11.19  (5.66) | -45.21  (16.12) | 59.62  (63.94) | -32.17  (14.22) | 0.93 | 44.02 | 4.04 | 4.04 | 3.99 | 3.31 | 4.41 | 1.98 | 4.89 |
| Chile | 73.36  (20.62) | 101.91  (90.9) | -48.9  (18.13) | -26.72  (14.96) | 19.38  (7.09) | -44.60  (19.49) | -51.72  (15.6) | -31.94  (14.93) | 0.85 | 22.77 | 24.28 | 3.78 | 4.33 | 3.64 | 3.68 | 2.25 | 5.06 |
| Colombia | 75.26  (15.32) | 107.84  (81.55) | -47.46  (19.15) | -22.19  (20.68) | 17.72  (8.15) | -43.58  (22.22) | -40.05  (17.34) | -31.50  (19.01) | 0.77 | 13.25 | 44.22 | 4.03 | 3.66 | 3.86 | 3.96 | 2.90 | 4.69 |
| Croatia | 50.04  (23.55) | 165.91  (281.48) | -16.98  (28.51) | -2.98  (16.84) | 5.34  (6.91) | -26.22  (23.02) | 82.88  (121.89) | -23.81  (15.54) | 0.85 | 22.67 | 73.73 | 4.05 | 4.02 | 4.00 | 4.14 | 2.55 | 4.60 |
| Denmark | 54.09  (11.98) | 93.86  (144.81) | -5.37  (16.78) | -3.26  (6.02) | 5.78  (5.02) | -27.34  (15.95) | 110.08  (92.06) | -24.32  (14.52) | 0.94 | 46.68 | 136.52 | 3.91 | 4.16 | 3.19 | 4.54 | 1.86 | 5.03 |
| Egypt | 61.71  (22.02) | 4.26  (4.54) | -28.36  (18.17) | 13.51  (19.6) | 6.89  (5.31) | -21.70  (20.55) | -9.86  (20.97) | -15.06  (13.67) | 0.71 | 10.55 | 98.00 | 3.66 | 3.98 | 4.45 | 3.20 | 2.20 | 4.42 |
| Estonia | 43.76  (17.73) | 73.96  (130.79) | -11.36  (18.71) | 1.22  (10.22) | 4.72  (6.11) | -15.95  (15.87) | 60.65  (62.88) | -24.01  (13.37) | 0.89 | 29.48 | 31.03 | 3.79 | 4.31 | 3.81 | 3.80 | 2.04 | 4.58 |
| Fiji | 60.56  (13.67) | 0.18  (0.71) | -9.56  (14.2) | -1.84  (11.41) | 9.54  (4.46) | -25.83  (11.58) | -10.29  (6.94) | -11.56  (10.73) | 0.74 | 8.70 | 49.56 | 3.77 | 3.83 | 4.33 | 3.53 | 2.58 | 4.67 |
| Finland | 44.05  (15.33) | 20.74  (23.98) | -16.11  (15.25) | -1.61  (6.53) | 5.3  (4.9) | -34.48  (13.67) | 96.04  (82.93) | -25.88  (12.58) | 0.94 | 40.59 | 18.14 | 3.66 | 4.34 | 3.37 | 4.45 | 1.80 | 4.90 |
| France | 61.41  (19.3) | 131.07  (208.8) | -31.01  (26.87) | -7.48  (15.92) | 10.28  (8.86) | -32.42  (24.44) | 26.79  (72.9) | -31.02  (17.5) | 0.90 | 38.61 | 122.58 | 3.72 | 4.21 | 3.20 | 4.76 | 2.21 | 5.05 |
| Georgia | 69.02  (18.83) | 214.40  (344.94) | -27.49  (25.14) | -7.28  (18.34) | 6.11  (8.17) | -21.91  (21.46) | 6.33  (30.73) | -28.54  (16.68) | 0.81 | 9.75 | 65.03 | 3.73 | 4.09 | 4.12 | 3.74 | 2.46 | 4.66 |
| Germany | 59.23  (15.09) | 66.28  (100.98) | -21.74  (20.04) | -2.25  (8.41) | 6.76  (4.79) | -26.00  (15.34) | 55.46  (47.87) | -21.35  (12.36) | 0.95 | 45.23 | 237.02 | 3.93 | 4.54 | 3.10 | 4.52 | 1.82 | 5.01 |
| Ghana | 51.38  (12.42) | 5.85  (8.04) | -9.77  (14.91) | 5.64  (18.72) | 11.40  (4.79) | -8.14  (19.04) | -12.18  (7.91) | -13.18  (8.88) | 0.61 | 4.23 | 126.72 | 4.12 | 3.53 | 4.27 | 3.19 | 2.68 | 4.73 |
| Greece | 64.58  (15.91) | 43.15  (69.6) | -25.33  (31.14) | 6.66  (11.9) | 6.92  (8.71) | -28.16  (24.87) | 58.16  (76.14) | -24.51  (17.03) | 0.89 | 24.57 | 83.48 | 4.25 | 4.40 | 3.41 | 4.16 | 1.83 | 4.84 |
| Hungary | 58.59  (14.13) | 111.19  (179.95) | -16.67  (20.09) | -4.32  (10.98) | 6.91  (6.08) | -24.23  (15.95) | 34.86  (44.58) | -24.33  (13.16) | 0.85 | 26.78 | 108.04 | 3.73 | 4.34 | 3.60 | 4.10 | 1.94 | 4.51 |
| India | 75.4  (21.24) | 23.01  (21.02) | -46.34  (23.38) | -9.22  (19.79) | 14.29  (7.24) | -32.52  (19.99) | -40.17  (18.57) | -29.27  (16.42) | 0.65 | 6.43 | 450.42 | 4.28 | 3.92 | 3.97 | 3.75 | 3.05 | 4.45 |
| Indonesia | 62.26  (10.11) | 9.04  (7.8) | -21.13  (10.71) | -4.44  (8.08) | 11.27  (4.02) | -36.27  (13.8) | -16.72  (12.31) | -22.50  (9.22) | 0.72 | 11.19 | 145.73 | 3.84 | 3.82 | 4.27 | 3.68 | 2.56 | 4.32 |
| Ireland | 66.55  (20.35) | 69.69  (114.58) | -38.80  (22.33) | -3.64  (9.09) | 14.10  (7.13) | -44.07  (16.01) | 14.10  (34.52) | -36.76  (15.57) | 0.96 | 67.34 | 69.87 | 4.04 | 3.77 | 3.41 | 4.30 | 2.09 | 4.90 |
| Italy | 73.21  (11.4) | 111.45  (166.01) | -31.68  (27.53) | -14.87  (16.68) | 10.39  (10.1) | -37.46  (22.86) | 11.10  (61.98) | -30.88  (17.3) | 0.89 | 35.22 | 205.86 | 3.81 | 4.62 | 3.46 | 4.11 | 1.60 | 5.27 |
| Japan | 36.47  (7.33) | 6.10  (7.29) | -12.61  (9.4) | 0.31  (2.41) | 7.07  (3.93) | -25.49  (11.32) | -2.75  (9.37) | -14.40  (9.98) | 0.92 | 39.00 | 347.78 | 4.06 | 4.21 | 3.49 | 4.27 | 2.65 | 4.36 |
| Jordan | 70.40  (21) | 95.14  (159.41) | -25.35  (22.35) | -5.71  (20.44) | 10.24  (7.67) | -60.08  (17.85) | -15.88  (25.05) | -28.41  (19.08) | 0.73 | 8.34 | 109.29 | 4.20 | 3.67 | 4.20 | 3.71 | 2.50 | 4.40 |
| Latvia | 50.84  (13.43) | 74.74  (146.83) | -11.68  (16.45) | 0.67  (10.1) | 3.97  (5.77) | -18.53  (16.32) | 58.66  (55.27) | -22.93  (11.99) | 0.87 | 25.06 | 31.21 | 3.75 | 4.46 | 3.83 | 3.85 | 1.80 | 4.32 |
| Malaysia | 62.58  (15.9) | 11.41  (17.54) | -33.92  (21.92) | -9.83  (15.19) | 14.29  (10.37) | -40.01  (21.68) | -23.28  (21.2) | -24.82  (16.9) | 0.81 | 26.81 | 96.25 | 3.91 | 3.65 | 4.35 | 3.57 | 2.25 | 4.41 |
| Mexico | 68.25  (19.66) | 35.81  (26.29) | -35.63  (14.68) | -9.63  (8.17) | 12.86  (4.95) | -39.55  (14.75) | -36.17  (12.07) | -26.44  (11.58) | 0.78 | 17.34 | 66.44 | 3.90 | 4.50 | 3.90 | 3.60 | 2.13 | 4.73 |
| Namibia | 53.29  (13.51) | 29.19  (46.9) | -19.19  (13.56) | -5.96  (11.71) | 8.16  (6.4) | -43.23  (14.95) | -17.54  (11.04) | -16.28  (15.32) | 0.65 | 9.54 | 3.08 | 4.06 | 3.74 | 4.04 | 3.66 | 2.53 | 4.48 |
| Nepal | 74.17  (22.01) | 27.45  (35.24) | -39.41  (23.43) | -22.36  (24.87) | 12.49  (6.83) | -30.30  (26.44) | -22.58  (18.66) | -29.97  (20.74) | 0.60 | 2.44 | 204.43 | 4.13 | 4.34 | 4.18 | 3.53 | 3.03 | 4.63 |
| Netherlands | 58.58  (16.76) | 154.26  (200.4) | -20.30  (17.33) | -4.43  (5.42) | 8.79  (4.37) | -42.57  (13.54) | 67.75  (66.86) | -26.66  (11.47) | 0.94 | 48.47 | 508.54 | 3.97 | 4.05 | 3.19 | 4.49 | 1.91 | 5.03 |
| Nigeria | 63.12  (18.98) | 1.39  (1.21) | -20.81  (15.59) | -10.64  (14.64) | 13.36  (6.93) | -10.14  (21.41) | -21.55  (14.54) | -17.77  (12.53) | 0.54 | 5.34 | 209.59 | 3.90 | 3.75 | 4.41 | 3.10 | 2.72 | 4.79 |
| Norway | 48.45  (15.9) | 29.86  (37.26) | -10.52  (16.07) | 3.83  (10.51) | 5.91  (5.11) | -29.73  (14.7) | 69.96  (66.13) | -26.84  (13.66) | 0.96 | 64.80 | 14.46 | 3.85 | 4.40 | 3.45 | 4.19 | 1.49 | 5.12 |
| Oman | 72.36  (23.32) | 78.51  (94.87) | -33.69  (14.47) | -23.24  (10.99) | 10.68  (6.59) | -45.42  (16.99) | -36.26  (11.93) | -25.68  (12.03) | 0.81 | 37.96 | 14.98 | 3.83 | 3.71 | 4.50 | 3.30 | 2.15 | 4.49 |
| Pakistan | 63.56  (17.85) | 6.91  (6.95) | -20.48  (21.56) | -5.33  (22.47) | 7.68  (6.07) | -13.71  (22.41) | -4.42  (15.26) | -19.48  (16.03) | 0.56 | 5.03 | 255.57 | 4.00 | 3.99 | 4.31 | 3.44 | 2.44 | 4.65 |
| Peru | 81.73  (13.57) | 100.20  (92.11) | -55.98  (20.64) | -34.37  (18.14) | 23.76  (8.99) | -55.63  (18.54) | -45.84  (17.37) | -43.74  (18.52) | 0.78 | 12.24 | 25.13 | 4.08 | 3.71 | 3.92 | 3.64 | 2.76 | 4.84 |
| Philippines | 74.34  (18.83) | 13.35  (12.31) | -49.25  (21.11) | -23.48  (18.12) | 21.60  (8.09) | -55.91  (19.45) | -30.81  (15.67) | -38.15  (18.09) | 0.72 | 7.60 | 351.87 | 3.76 | 4.04 | 4.03 | 3.48 | 2.68 | 4.59 |
| Poland | 56.99  (21.07) | 114.01  (187.96) | -19.91  (22.41) | -5.1  (15.01) | 6.34  (6.38) | -30.13  (18.27) | 45.79  (68.38) | -21.52  (12.28) | 0.88 | 27.22 | 124.03 | 3.84 | 3.86 | 3.86 | 3.82 | 2.51 | 4.48 |
| Portugal | 64.47  (12.94) | 137.87  (184.03) | -30.51  (21.58) | -9.94  (14.82) | 13.33  (8.36) | -42.51  (17.74) | 5.59  (49.47) | -29.47  (15.77) | 0.86 | 27.94 | 112.37 | 4.11 | 4.27 | 3.43 | 4.08 | 1.89 | 5.21 |
| Romania | 58.68  (18.99) | 107.15  (137.81) | -25.79  (20.86) | -7.12  (14.55) | 4.99  (6.57) | -29.26  (18.17) | -4.85  (31.48) | -25.13  (13.46) | 0.83 | 23.31 | 85.13 | 4.06 | 4.11 | 3.78 | 4.03 | 2.00 | 4.48 |
| Senegal | 47.74  (19.17) | 3.73  (3.01) | -22.94  (11.24) | -7.74  (15.02) | 8.45  (5.02) | -28.30  (14.26) | -17.64  (8.63) | -19.65  (9) | 0.51 | 2.47 | 82.33 | 3.74 | 3.58 | 4.45 | 3.14 | 2.63 | 4.92 |
| Serbia | 63.28  (19.58) | 191.47  (315.72) | -22.03  (23.3) | -3.80  (17.08) | 5.13  (7.65) | -25.50  (22.39) | 5.97  (34.06) | -27.61  (17.05) | 0.81 | 14.05 | 80.29 | 4.03 | 3.96 | 3.57 | 4.21 | 1.61 | 4.44 |
| Singapore | 52.17  (13.12) | 33.29  (46.64) | -28.52  (18.87) | -4.99  (8.62) | 20.53  (10.19) | -36.19  (15.95) | -26.01  (20.2) | -27.26  (18.08) | 0.94 | 85.54 | 7915.73 | 3.88 | 3.76 | 4.00 | 3.58 | 2.82 | 4.60 |
| Slovakia | 55.00  (17.68) | 174.52  (354.7) | -27.20  (26.57) | -8.79  (12.73) | 7.01  (6.37) | -28.99  (18.86) | 48.8  (59.14) | -24.82  (13.3) | 0.86 | 30.16 | 113.13 | 3.83 | 4.47 | 3.82 | 3.64 | 2.00 | 4.58 |
| Slovenia | 61.22  (19.97) | 204.01  (333.81) | -31.98  (29.1) | -23.28  (14.35) | 8.41  (8.57) | -26.41  (22.4) | 30.33  (56.42) | -28.15  (15.09) | 0.92 | 31.40 | 102.62 | 3.71 | 4.45 | 3.71 | 4.30 | 1.62 | 4.56 |
| South Africa | 65.24  (19.33) | 60.60  (68.09) | -28.26  (20.24) | -10.22  (15.36) | 16.03  (7.98) | -42.92  (19.37) | -25.57  (12.37) | -28.83  (17.32) | 0.71 | 12.29 | 46.75 | 3.89 | 3.86 | 4.03 | 3.67 | 2.59 | 4.52 |
| Spain | 64.05  (17.51) | 131.24  (193.62) | -36.93  (26.65) | -12.70  (18.56) | 10.14  (9.11) | -37.82  (22.41) | -2.28  (41.05) | -30.54  (18.95) | 0.90 | 34.27 | 93.11 | 3.80 | 4.47 | 3.31 | 4.33 | 1.84 | 5.23 |
| Sweden | 56.11  (14.33) | 133.28  (348.62) | -10.13  (10.4) | -1.50  (4.41) | 5.97  (3.55) | -27.47  (11.47) | 92.58  (90.71) | -23.41  (11.7) | 0.95 | 46.95 | 24.72 | 3.81 | 4.46 | 3.12 | 4.67 | 1.83 | 4.90 |
| Switzerland | 49.90  (14.69) | 168.27  (350.05) | -27.11  (22.12) | -3.47  (6.78) | 8.14  (6.29) | -24.99  (14.62) | 34.91  (43.93) | -23.95  (12.77) | 0.96 | 57.41 | 214.24 | 3.86 | 4.17 | 3.19 | 4.64 | 2.24 | 4.99 |
| Thailand | 52.92  (17.93) | 0.37  (1.04) | -15.19  (14.71) | -0.62  (7.38) | 6.11  (5.63) | -30.96  (13.28) | -21.73  (12.77) | -14.84  (8.73) | 0.78 | 16.28 | 135.13 | 3.88 | 3.84 | 4.02 | 3.83 | 3.23 | 4.29 |
| Turkey | 64.15  (11.29) | 56.33  (98.54) | -34.71  (20.15) | -1.43  (14.58) | 8.61  (8.54) | -28.86  (21.92) | 7.82  (39.38) | -25.77  (16.11) | 0.82 | 25.13 | 104.91 | 3.98 | 4.23 | 3.77 | 3.91 | 2.97 | 4.77 |
| Uganda | 75.04  (15.98) | 2.66  (3.98) | -34.13  (17) | -19.70  (17.57) | 15.54  (7.1) | -40.09  (18.5) | -14.14  (8.8) | -20.02  (14.98) | 0.54 | 1.70 | 213.76 | 4.02 | 3.97 | 4.23 | 3.24 | 2.99 | 4.39 |
| Venezuela | 84.18  (4.01) | 13.42  (12.09) | -38.10  (14.28) | -16.55  (15.65) | 15.25  (3.26) | -37.95  (17.97) | -33.90  (12.74) | -25.09  (12.09) | 0.71 | 16.75 | 36.25 | 4.01 | 3.99 | 3.74 | 3.85 | 2.09 | 4.77 |
| Yemen | 38.97  (19.54) | 0.27  (0.46) | 10.33  (17.22) | 21.64  (18.79) | 4.42  (1.94) | 5.16  (13.98) | 12.50  (21.02) | -2.75  (10.6) | 0.47 | 1.48 | 53.51 | 3.79 | 3.70 | 4.63 | 3.06 | 2.28 | 4.73 |
| Zimbabwe | 75.28  (9.72) | 3.49  (5.87) | -15.85  (21.9) | -2.46  (23.27) | 18.12  (11.4) | -25.40  (28.21) | -0.57  (26.14) | -13.37  (20.5) | 0.57 | 1.90 | 42.73 | 4.19 | 3.62 | 4.04 | 3.70 | 2.67 | 4.30 |
| GSI: government stringency index; DNC: daily new cases per million people; RR: retail and recreation; GP: grocery and pharmacy; RE: residential; TS: transit stations; PA: parks; WO: workplace; GDP: GDP per capita; PD: population density; MA: mastery; HA: harmony; EM: embeddedness; AU: autonomy; HI: hierarchy; EG: egalitarianism. | | | | | | | | | | | | | | | | | |

In this study, we omitted mobility and only allowed intercepts to vary across countries to build a null model. The ICC of RE model was >0 (RE: ICC = 0. 337). The subsequent multilevel model analysis is reasonable. The finding is consistent with the hypothesis that the GSI negatively predicts mobility (See **Table 2**). A more stringent policy predicted RE positively (*β* = 0.654, *p* < 0.001).

| **Table 2** Multilevel regression results for GSI on mobility | | | | | | |
| --- | --- | --- | --- | --- | --- | --- |
| Dependent variable | **RE** |  |  | **PS** |  |  |
|  | B | se | β | B | se | β |
| **Intercept** | 15.586 | 9.198 |  | -12.545 | 27.423 |  |
| **GDP** | 0.639 | 2.078 | 0.006 | -5.817 | 6.196 | -0.246 |
| **HDI** | -14.732 | 15.319 | -0.228 | 67.191 | 45.677 | 0.381 |
| **PD** | 0.001 | 0.001 | 0.094 | -0.001 | 0.002 | -0.041 |
| **GSI** | 0.329 | 0.019 | 0.654^***^ | -0.786 | 0.046 | -0.572^***^ |
| ^†^ p < 0.01. * p < 0.05, ** p < 0.01, *** p < 0.001 | | | | | | |

Next, we examined whether DNC moderated the relationship between GSI and mobility. In Table 3, The positive relationship between GSI and RE was enhanced by DNC (*β* = 0.132, *p* = 0.017).

| **Table 3** Multilevel regression results for GSI*DNC on mobility | | | | | | |
| --- | --- | --- | --- | --- | --- | --- |
| Dependent variable | **RE** |  |  | **PS** |  |  |
|  | **B** | **se** | **β** | **B** | **se** | **β** |
| **Intercept** | 15.011 | 8.804 |  | -6.017 | 24.684 |  |
| **GSI** | 0.379 | 0.019 | 0.754*** | -0.935 | 0.061 | -0.680*** |
| **DNC** | -0.384 | 0.157 | -0.071* | 0.427 | 0.590 | 0.028 |
| **GDP** | 0.562 | 1.986 | 0.064 | -2.538 | 5.565 | -0.107 |
| **HDI** | -13.370 | 14.630 | -0.214 | 20.690 | 41.004 | 0.117 |
| **PD** | 0.001 | 0.001 | 0.169* | -0.002 | 0.002 | -0.081 |
| **GSI*DNC** | 0.033 | 0.013 | 0.132* | -0.114 | 0.031 | -0.169*** |
| ^†^ p < 0.01. * p < 0.05, ** p < 0.01, *** p < 0.001 | | | | | | |

We also employed the Johnson-Neyman (J-N) technique to identify the region of significance and slope change trend of the conditional effect of GSI on mobility. In **Fig 1**, as DNC increased, the positive relationship between GSI and RE was stronger.


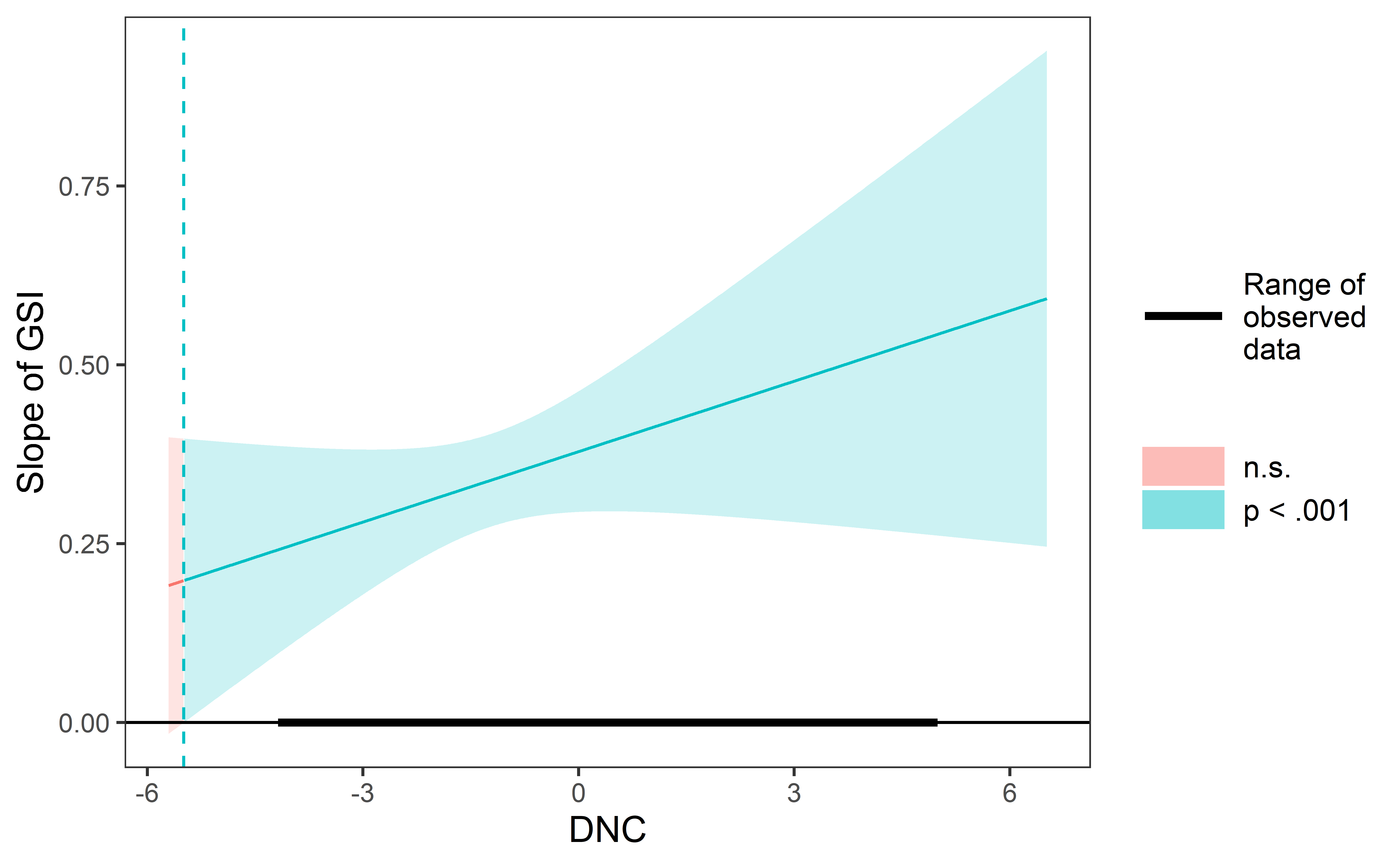


**Fig. 1** Conditional effect of GSI on mobility (RE) as a function of DNC

To better explain the interaction between the GSI and DNC on mobility, we calculated the actual mobility in different GSI and DNC situations over different periods (refer to **Fig 2**).


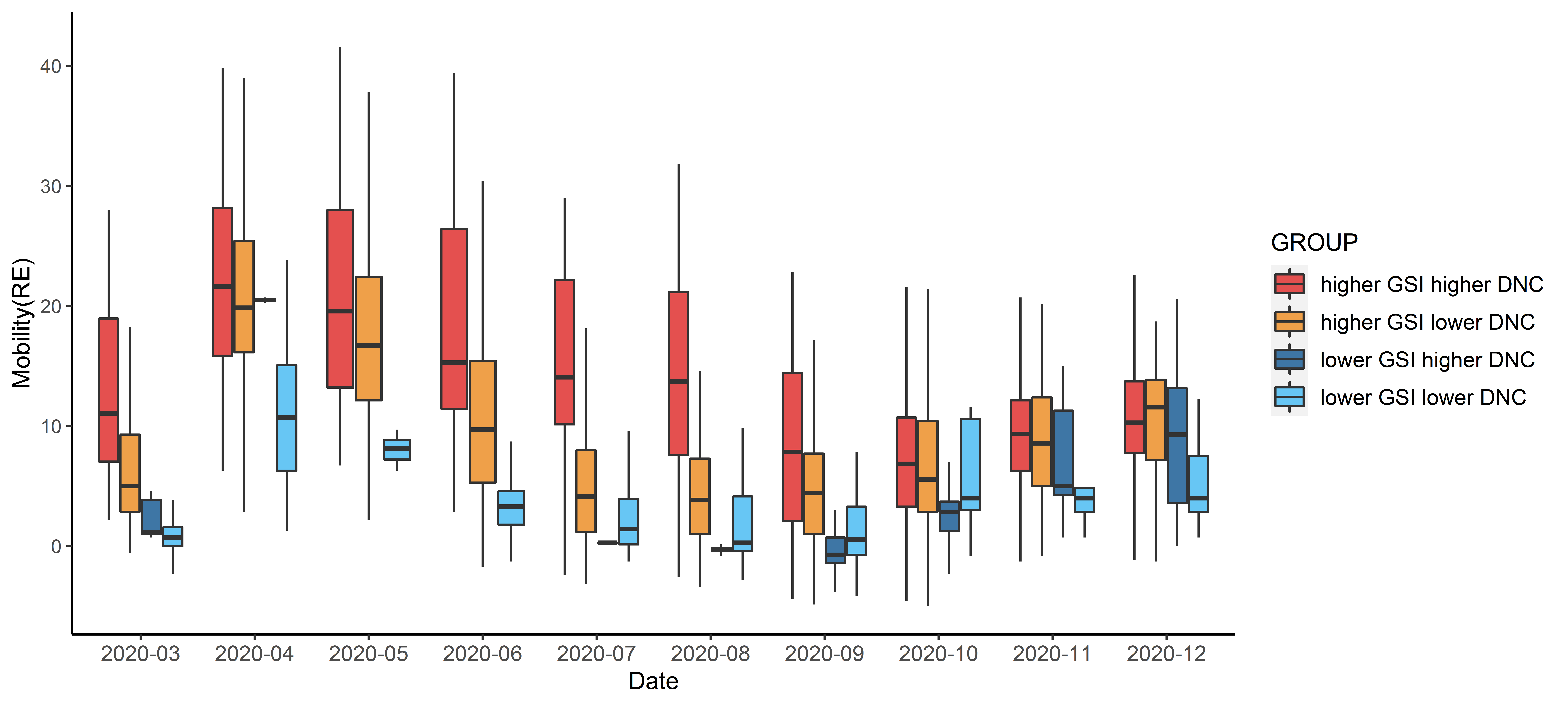


**Fig. 2** Average RE under GSI and DNC conditions in different periods

The three-way interaction term of embeddedness (*β* = -0.087, *p* = 0.093), autonomy (*β* = 0.095, *p* = 0.056), and hierarchy (*β* = -0.088, *p* = 0.084) were marginally significant and exhibited a trend similar to the model with PS as the dependent variable (**Table 4**).

| **Table 4** Moderated moderation model of RE (the coefficients of the interaction terms) | | | | | | |
| --- | --- | --- | --- | --- | --- | --- |
| **Variables** | **Cultural Values** | | | | | |
|  | **Embeddedness** | **Autonomy** | **Hierarchy** | **Egalitarianism** | **Mastery** | **Harmony** |
| **GSI** | 0.756*** | 0.755*** | 0.754*** | 0.754*** | 0.753*** | 0.755*** |
| **GSI*DNC** | 0.134* | 0.133* | 0.133* | 0.133* | 0.132* | 0.134* |
| **GSI*VALUE** | -0.080^†^ | 0.096^†^ | -0.052 | 0.061 | -0.110* | 0.027 |
| **DNC*VALUE** | -0.046 | 0.036 | -0.054^†^ | 0.042 | -0.079** | 0.054^†^ |
| **GSI*DNC*VALUE** | -0.087^†^ | 0.095^†^ | -0.088^†^ | 0.064 | -0.008 | 0.042 |
| ^†^ p < 0.01. * p < 0.05, ** p < 0.01, *** p < 0.001 | | | | | | |

Using the Johnson-Neyman (J-N) technique, we can observe how the moderation role of DNC changed under different cultural values. In **Fig. 3**, we plot the conditional effect of GSI on RE as a function of DNC under different embeddedness values (mean ±1SD). Autonomy J-N plot can be found in **Fig. 4**. Hierarchy J-N plot can be found in **Fig. 5**. Egalitarianism J-N plot can be found in **Fig. 6**. Mastery J-N plot can be found in **Fig. 7**. Harmony J-N plot can be found in **Fig. 8**.


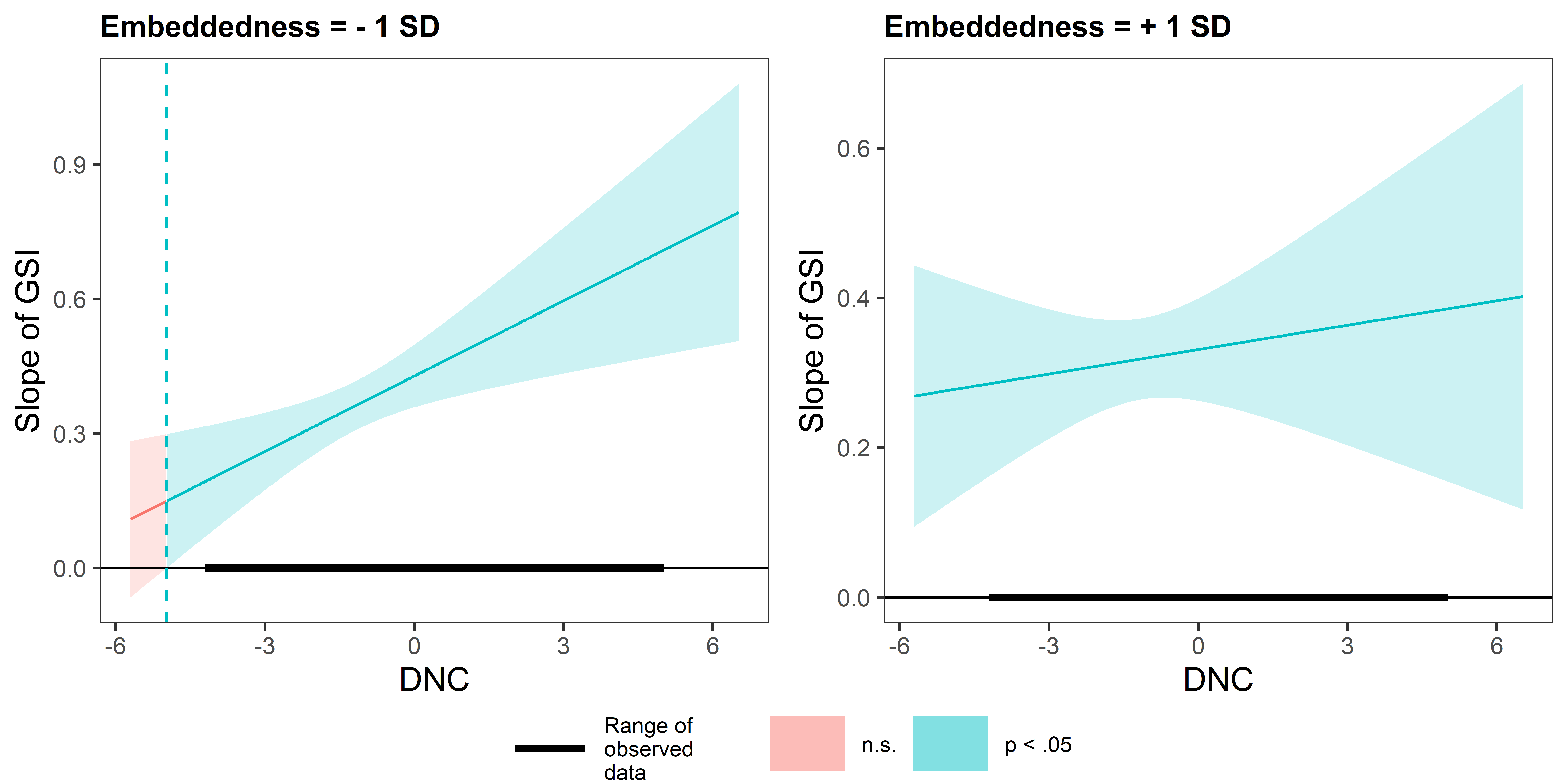


**Fig. 3** Conditional effect of GSI on RE as a function of DNC under different embeddedness values.


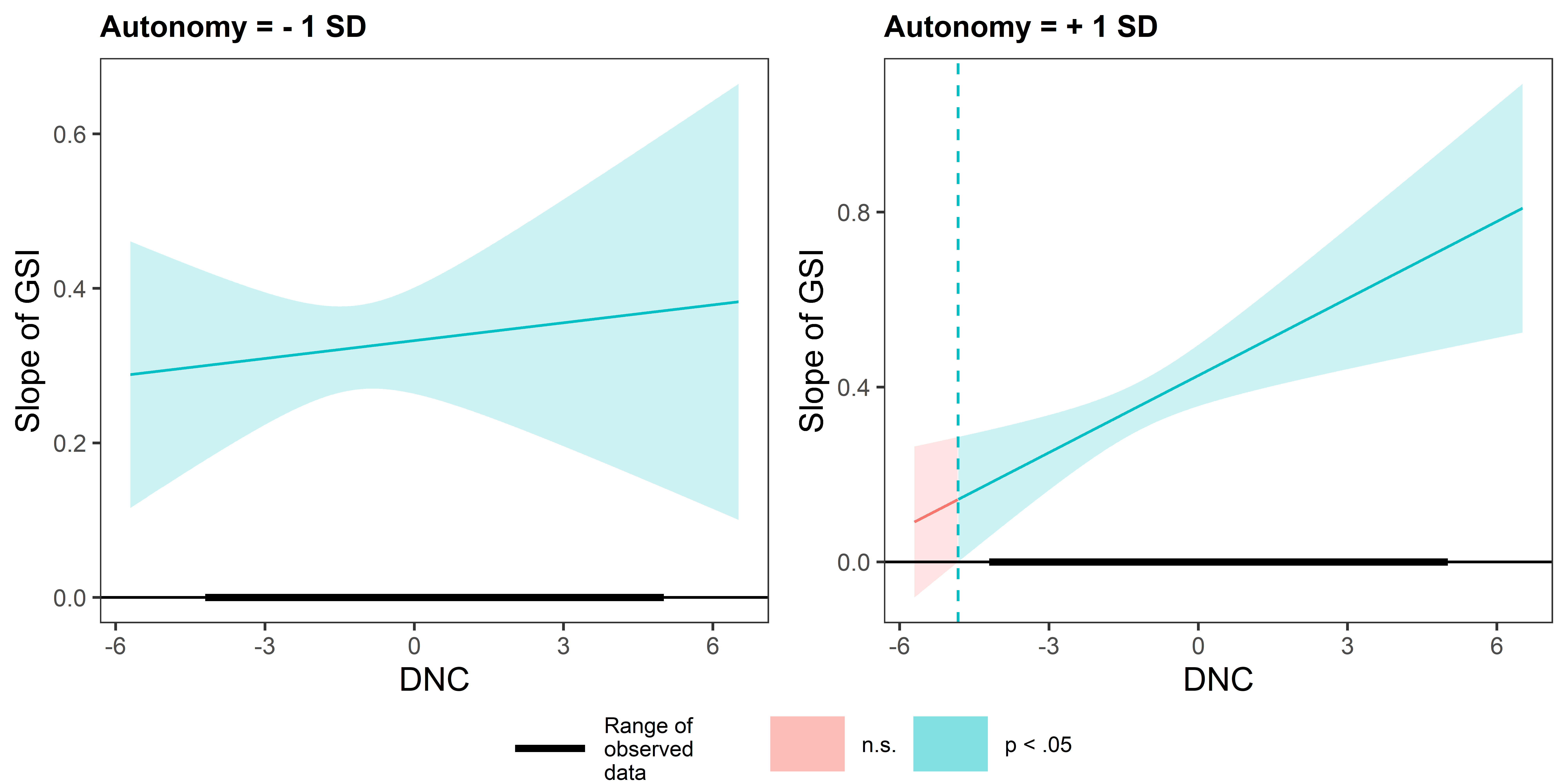


**Fig. 4** Conditional effect of GSI on RE as a function of DNC under different autonomy values.


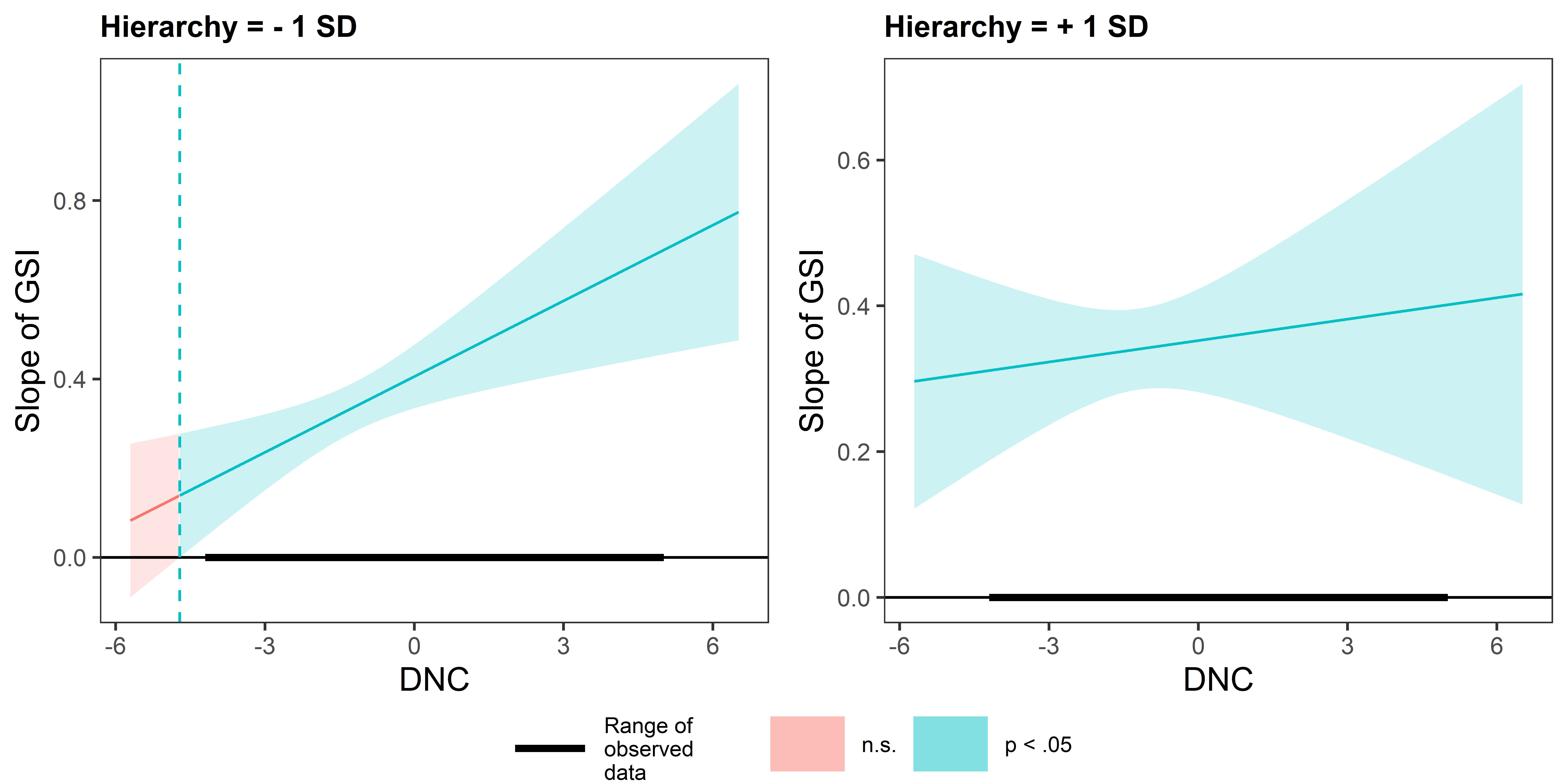


**Fig. 5** Conditional effect of GSI on RE as a function of DNC under different hierarchy values.


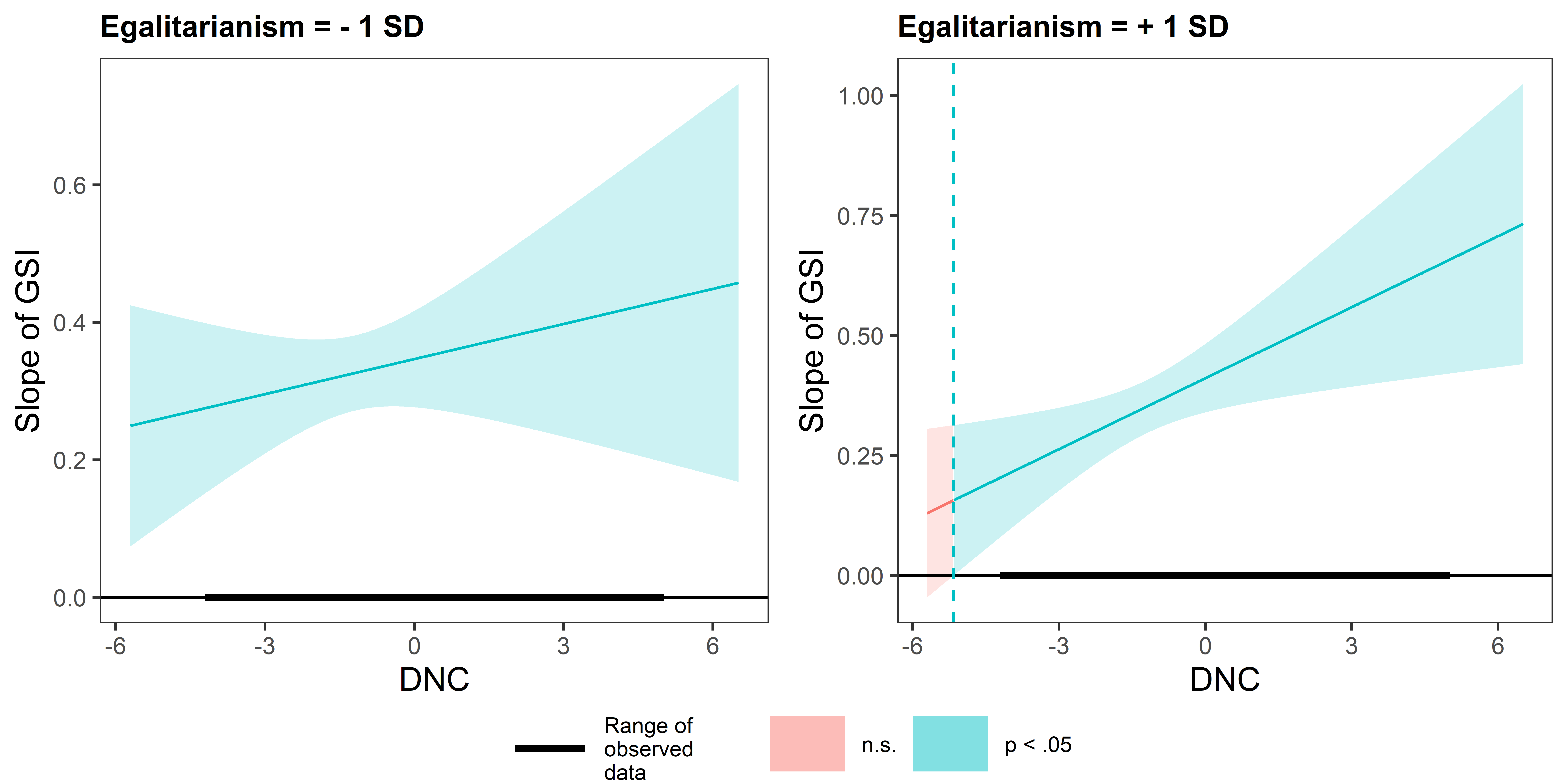


**Fig. 6** Conditional effect of GSI on RE as a function of DNC under different egalitarianism values.


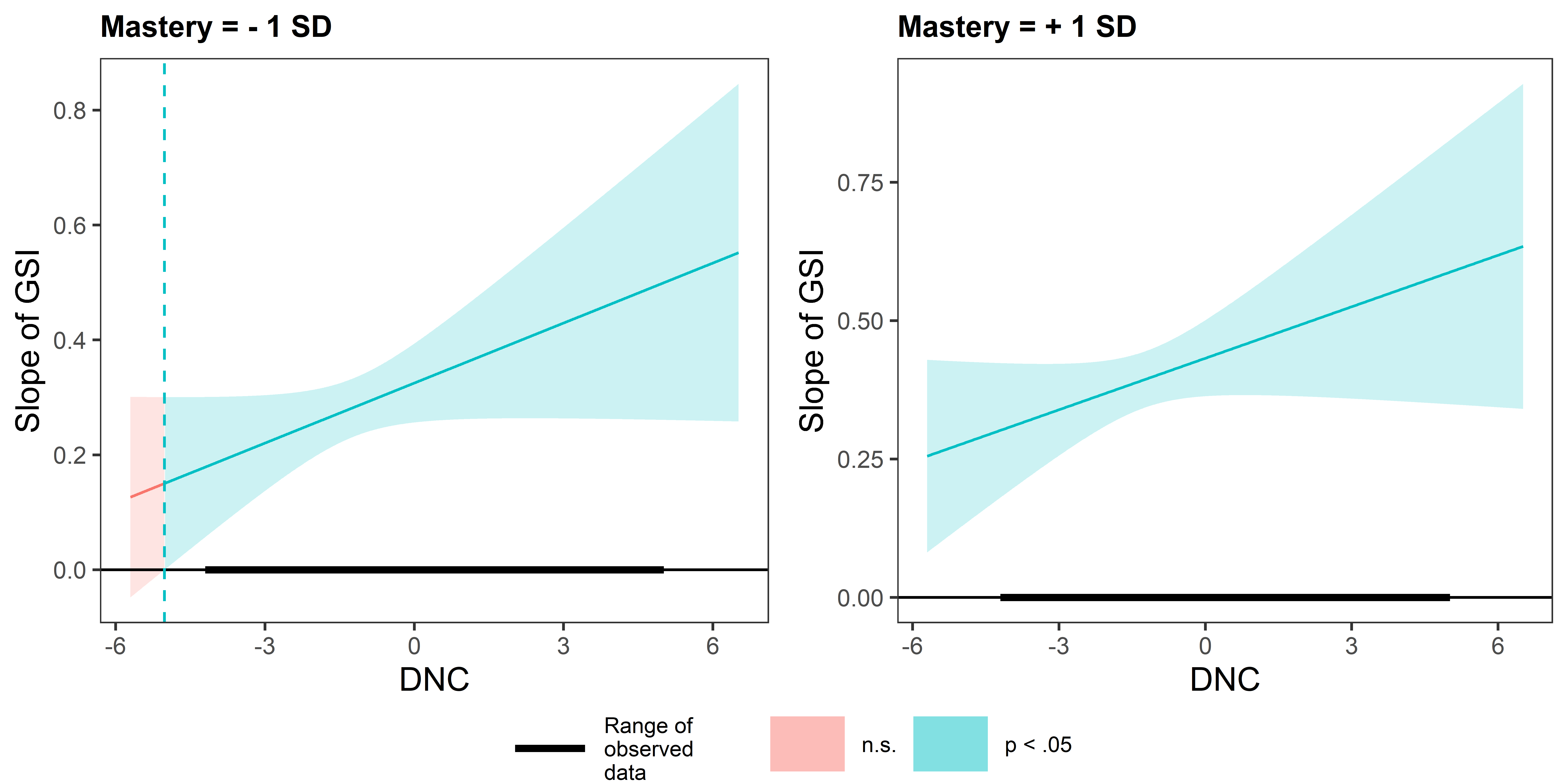


**Fig. 7** Conditional effect of GSI on RE as a function of DNC under different mastery values.


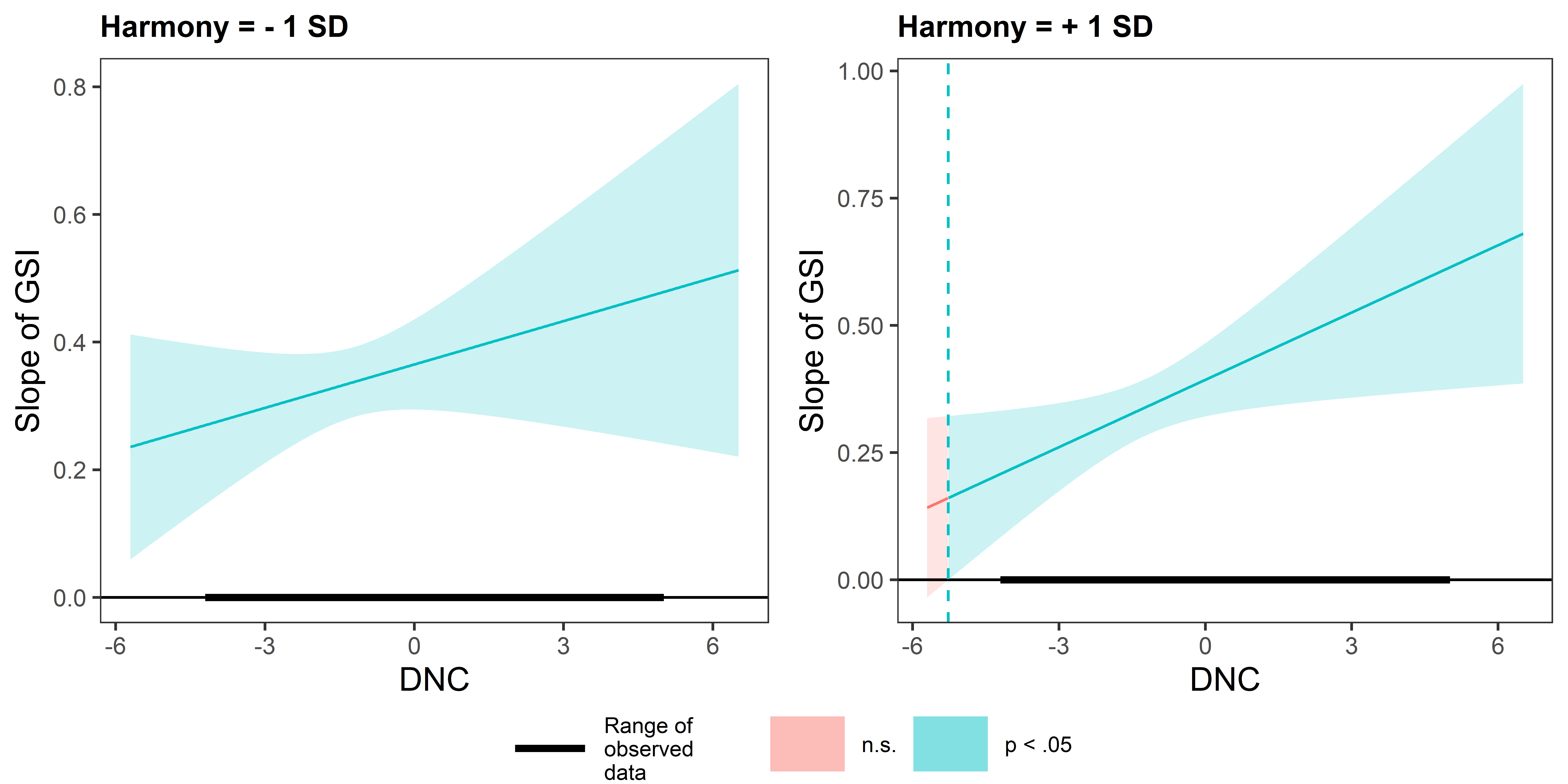


**Fig. 8** Conditional effect of GSI on RE as a function of DNC under different harmony values.

To better comprehend the role of cultural values, as a supplementary analysis, we also categorized the countries into three equal segments based on their values and assessed the moderating effect of DNC in higher-value and lower-value countries. In countries that prioritize embeddedness, the moderating effect is not significant (*β_GSI*DNC_* = -0.046, *p* = 0.709). In countries that do not prioritize embeddedness, the moderating effect is significant (*β_GSI*DNC_* = 0.318, *p* < 0.001). The effect of hierarchy and mastery is similar to that of embeddedness. Higher hierarchy: *β_GSI*DNC_* = 0.086, *p* = 0.543; Lower hierarchy: *β_GSI*DNC_* = 0.335, *p* < 0.001. Higher mastery: *β_GSI*DNC_* = 0.161, *p* = 0.224; Lower mastery: *β_GSI*DNC_* = 0.217, *p* = 0.004. In countries that prioritize autonomy, the moderating effect is significant (*β_GSI*DNC_* = 0.311, *p* < 0.001). In countries that do not prioritize autonomy, the moderating effect is insignificant (*β_GSI*DNC_* = -0.056, *p* = 0.700). The effect of egalitarianism and harmony is similar to that of autonomy values. Higher egalitarianism: *β_GSI*DNC_* = 0.322, *p* < 0.001; Lower egalitarianism: *β_GSI*DNC_* = 0.145, *p* = 0.369. Higher harmony: *β_GSI*DNC_* = 0.271, *p* < 0.001; Lower harmony: *β_GSI*DNC_* = 0.146, *p* = 0.199.
